# Integrative analysis and experimental validation identify the role of CD44 and Nucleolin in regulating gliogenesis following spinal cord injury

**DOI:** 10.1186/s13619-025-00253-x

**Published:** 2025-08-13

**Authors:** Ming Shi, Yazhou Sun, Lu Ding, Xinyue Li, Qi Xu, Fuxin Wei, Tianshun Gao, David Y. B. Deng

**Affiliations:** 1 Scientific Research Center, The Seventh Affiliated Hospital of Sun Yat-Sen University, Shenzhen, 518107 China; 2 Department of Orthopedic Surgery, The Seventh Affiliated Hospital of Sun Yat-Sen University, Shenzhen, 518107 China; 3 Big Data Center, The Seventh Affiliated Hospital of Sun Yat-Sen University, Shenzhen, 518107 China; 4 Shenzhen Key Laboratory of Bone Tissue Repair and Translational Research, Shenzhen, 518107 China

**Keywords:** Spinal cord injury, ScRNA-seq/snRNA-seq analysis, Ligand-receptor interaction, Inflammation, Gliogenesis

## Abstract

Spinal cord injury (SCI) triggers a complex cascade of cellular and molecular responses, yet the complex cellular communication remains incompletely understood. This study explored how intercellular communication contributes to the activation of microglia and astrocytes after SCI. Here, we integrated four datasets using single-cell RNA sequencing (scRNA-seq) or single-nucleus RNA sequencing (snRNA-seq) and constructed a comprehensive cellular atlas of the injured spinal cord. Transcriptomic changes in microglia and astrocytes were analyzed. We identified CD44 as a key receptor in SPP1-mediated microglial activation, which represented a subpopulation involved in inflammatory response in microglia. We defined a gliogenesis subpopulation of astrocytes that emerged at 3 dpi, which became the predominant cell type in the injured spinal cord. These astrocytes highly expressed the Nucleolin (*Ncl*) gene and interacted via the Pleiotrophin (*Ptn*) signaling pathway, which is associated with astrocyte proliferation. To validate these findings, we utilized a crush injury model. Flow cytometry of isolated microglia and astrocytes confirmed the upregulation of CD44 in microglia and NCL in astrocytes in response to SCI. In vivo results confirmed that the CD44 positive microglia accumulated and PLA results further confirmed the combination of SPP1 with CD44. In parallel, the upregulated expression of NCL in astrocytes facilitated their proliferation, underscoring the role of the NCL receptor in gliogenesis after SCI. In vitro validation demonstrated that exogenous SPP1 upregulates CD44 expression by promoting the phosphorylation of p65 and activating the NF-κB pathways in BV2 microglia, and that high expression of IL-6 indicates the activation of inflammation. PTN may enhance NCL expression and thus facilitates astrocyte proliferation. Collectively, our study identified key receptors that regulated inflammation responses and gliogenesis. Targeting the CD44 and NCL receptors may provide promising therapeutic strategies to modulate inflammation and promote tissue repair after SCI.

**Supplementary Information:**

The online version contains supplementary material available at 10.1186/s13619-025-00253-x.

## Background

Spinal cord injury (SCI) is a devastating condition that initiates a cascade of cellular and molecular responses, leading to neural inflammation, synaptic dysfunction (Ahuja et al. 2017), cell death (O'Shea et al. 2017), scar formation (Dorrier et al. 2021), and ultimately impacting tissue repair and recovery. While these processes have been studied extensively, the specific roles of cell–cell interactions remain poorly understood. Recent advances in scRNA-seq have provided a powerful tool to dissect the cellular heterogeneity and intercellular communication in SCI (Milich et al. 2021b; Rosenberg et al. 2018). However, despite these advancements, the phenotypic diversity within cell types and the mechanisms by which they interact during the repair process are still not fully understood. A deeper investigation into these interactions, particularly ligand-receptor signaling, is crucial for uncovering the molecular drivers of inflammation and tissue remodeling after SCI.

Cellular behavior is governed by both intracellular regulatory networks and the extracellular signaling environment, which together determine cellular functions (Shao et al. 2020). One primary form of intercellular communication is ligand–receptor interactions, which regulate various aspects of the injury response, including autocrine, paracrine, and endocrine signaling (Qian et al. 2024). Cell–cell interactions are critical for maintaining normal spinal cord function and play a key role in tissue repair after injury. By integrating gene expression data with computational methods (Armingol et al. 2021), key ligand-receptor pairs that mediate critical processes such as inflammation can be identified. For example, Brennan et al. demonstrated that CCL3/CCL4-CCR5 interactions between microglia and monocyte-derived macrophages (MDMs) are crucial during the acute phase of SCI. However, excessive activation of this signaling pathway can lead to pathological consequences (Brennan et al. 2022). Other ligand-receptor interactions, such as Psap-Gpr37l1, influence astrocyte survival by protecting against oxidative stress (Meyer et al. 2013), while Cadm1-Cadm1 promote proliferation and adhesion to extracellular matrix proteins. Additionally, CellChat analyses also revealed that microglia normally produce ligands like SPP1, which can promote central nervous system (CNS) regeneration and neuroprotection (Anderson et al. 2018). These findings underscore the importance of specific signaling molecules and suggest potential therapeutic targets for enhancing neuroprotection and regeneration following SCI.

While previous studies have examined cellular responses, few have specifically addressed the role of ligand-receptor signaling in modulating inflammation and gliogenesis after SCI. This gap limits a comprehensive understanding of how these interactions precisely influence cellular behaviors such as inflammation, glia scar formation, and tissue regeneration. Given the importance of ligand-receptor interactions in coordinating intercellular communication and functional outcomes in the injury microenvironment, it is essential to investigate these mechanisms in greater detail. Focusing on the specific ligand-receptor pairs that control cell fate, proliferation, and functional recovery will not only deepen our understanding of SCI pathology but also reveal novel therapeutic targets to enhance regenerative outcomes.

Hence, in this study, we first integrated an extensive dataset, capturing multiple time points post-SCI to map intricate cellular dynamics. And then we focused specifically on microglia and astrocytes to refine the functional characterization of distinct cellular subsets, attributing unique roles based on their temporal activity, which are crucial for understanding the pathophysiological changes following SCI. Through a detailed analysis of receptor-ligand interactions, we explored the SPP1-CD44 signaling axis, illustrating the modulation of microglia activation via the CD44 receptor, shedding new light on the inflammatory response. Furthermore, we identified that the upregulation of NCL receptors in astrocytes was critical for triggering the activation and proliferation of reactive astrocytes. Finally, we employed a crush injury model to validate the spatial distribution of CD44 receptors in microglia in vivo, alongside comprehensive assessments of proliferation and NCL receptor expression using flow cytometry and immunofluorescence. In conclusion, our findings emphasize the pivotal roles of CD44 and NCL in regulating immune responses mediated by microglia and promoting astrocyte proliferation after SCI, suggesting novel therapeutic avenues for enhancing repair and regeneration.

## Results

### Cellular heterogeneity and their transcriptional responses

To investigate cellular heterogeneity and transcriptional dynamics in SCI, we integrated datasets from four studies (Table S1).

To assess dataset consistency, we analyzed nFeature_RNA and nCount_RNA distributions, which showed comparable sequencing depth and gene complexity across datasets (Fig. S1). The relative proportions of specific cell types, such as neurons, varied across datasets, reflecting experimental protocol-specific differences. The split UMAP clustering showed that all major cell-type identities were consistently preserved. Additionally, the Venn diagram indicated a 30% overlap in highly variable genes (HVGs), highlighting a conserved core set of transcriptional signatures shared across datasets.

Our primary objective was to construct a comprehensive atlas of the major spinal cord cell types across various time points. To achieve this, the filtered high-quality data were integrated with the RPCA to eliminate batch effects, harmonize the datasets, resulting in a unified dataset of 218,512 cells and nuclei. We performed clustering and identified highly expressed genes in each cluster. The original cell type annotations were preserved where available, and clusters lacking annotations were inferred based on literature-reported marker genes.

**Fig. 1:**
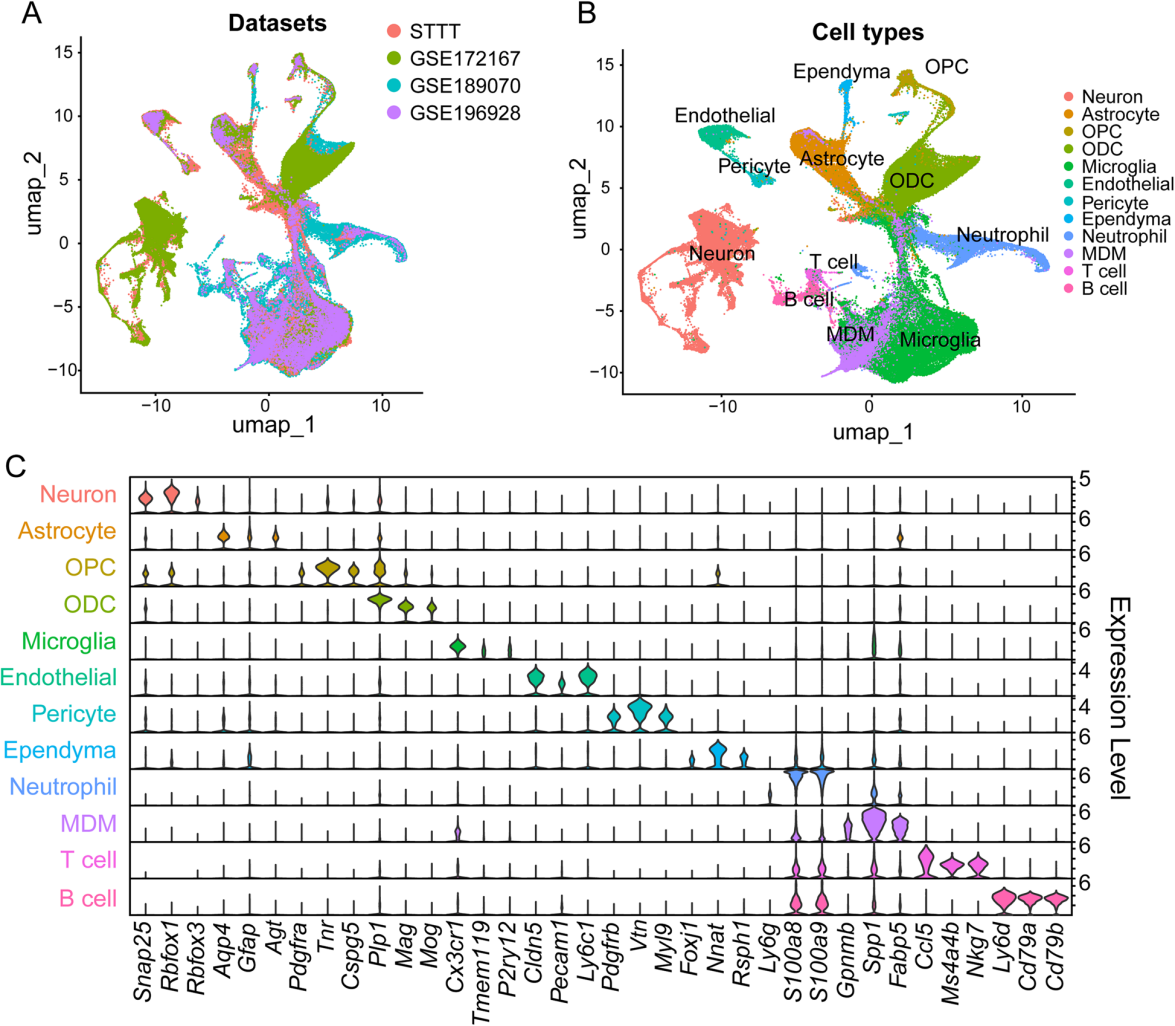
Integration of four independent studies data reveals the major cell types in the spinal cord. **A** UMAP projection of four SCI datasets, a total of 218,512 cells/nucleus in the final dataset. **B** UMAP plot depicted the distribution of 12 major cell types in the spinal cord, exhibiting distinct spatial separation, reflecting the heterogeneous response to injury. **C** Violin plots showing the expression levels of marker genes for specific cell types, illustrating the variability in gene expression profiles among cell types

The UMAP (Uniform Manifold Approximation and Projection) plot (Fig. 1A) illustrates the clustering of various cell populations across the integrated datasets. Twelve distinct clusters corresponding to major spinal cord cell types were identified (Fig. 1B). The cell type distribution revealed that neurons and astrocytes were predominant, while microglia and MDMs (monocyte-derived macrophages) were central to the acute injury response. Expression patterns of specific marker genes further validated cell-type identities (Fig. 1C).

Overall, these findings validate the successful integration of heterogeneous datasets, capturing both shared cellular traits and transcriptional signatures while accommodating dataset-specific differences. By harmonizing data across studies, we reduced inter-dataset variability, yielding a unified and reliable resource to explore the transcriptional and cellular landscape of SCI.

### Microglia activation was induced by SPP1 signaling pathway at 1 dpi

**Fig. 2:**
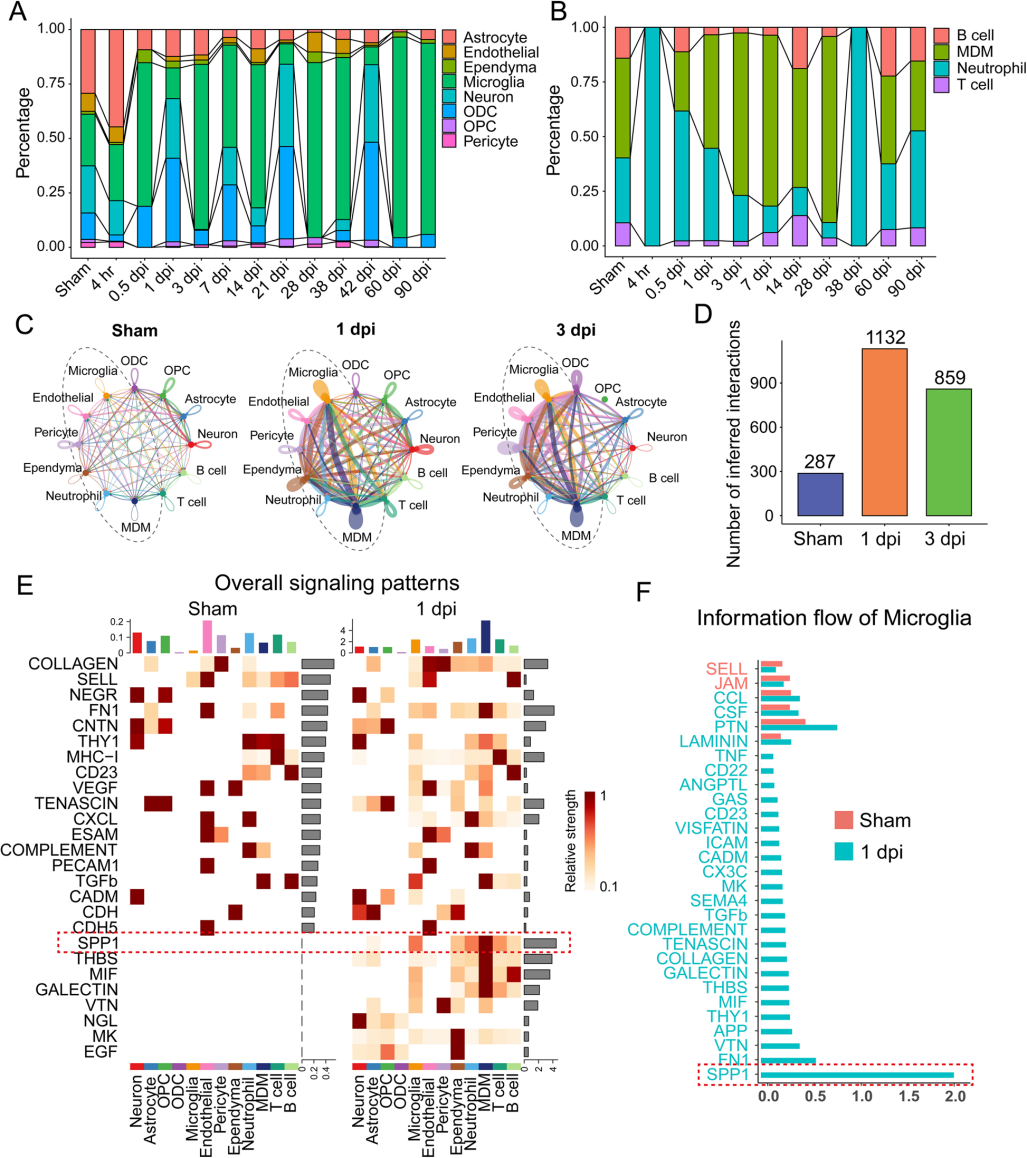
Dynamic cell portion changes and intercellular communication analysis revealed SPP1 signaling pathway was critical in microglia after SCI. **A**, **B** Stacked bar plots depicting changes in the relative abundance of major cell types in spinal cord (**A**) and peripheral immune cell populations (**B**) across various time points. Astrocytes, microglia, OPCs, and MDMs show marked shifts, particularly in the acute (1~3 dpi) and subacute phases of SCI, the absence of 42 dpi stems directly from the source data (GSE172167), where immune cell clusters were not identified or annotated at this specific time point in the original study. **C** Intercellular communication networks illustrate increased signaling complexity at 1 and 3 dpi compared to the sham condition. **D** Quantitative result of the total number of interactions in sham, 1 dpi, and 3 dpi samples, showing a significant increase in cell–cell interactions post-injury. **E** Heatmaps displaying the changes in signaling patterns for key cell types. SPP1 became prominent at 1 dpi. **F** Information flow of microglia indicated the SPP1 signal was significant

To investigate the temporal dynamics of cellular responses after SCI, we classified the 12 identified cell types into two major groups: spinal cord-resident cells (e.g., neurons, astrocytes, ependymal cells, and microglia), and peripheral immune cells (e.g., MDMs, neutrophils, B cells, and T cells) (Greenhalgh et al. 2020). Based on this classification, we examined overall trends in cell population dynamics after SCI. Neurons and astrocytes exhibited a general decline in abundance. Microglia remained relatively abundant at key time points following SCI (3dpi, 7dpi, 14dpi) (Fig. 2A). Among peripheral immune cells, neutrophils were the first to infiltrate the injury site, but their numbers rapidly declined to baseline within three days. MDMs exhibited an increase starting at 3 dpi (Fig. 2B). These observations suggested the involvement of microglia and macrophages in the acute inflammatory response following SCI.

To further investigate intercellular signaling complexity, we performed cell–cell communication analyses using CellChat (Jin et al. 2021b). At 1 dpi, the number and intensity of interactions increased sharply, with the strongest interactions occurring between MDMs and microglia (Fig. 2C, D). To assess changes in intercellular communication patterns, we compared the global signaling profiles of various cell types under Sham and 1 dpi conditions (Fig. 2E). Heatmaps depicting relative signaling strength revealed notable alterations in specific pathways. A marked increase in signaling activity was observed, particularly in injury- and inflammation-related pathways, such as SPP1and COMPLEMENT. Notably, the SPP1 signaling pathway showed strong activation at 1 dpi, mainly in microglia and other immune-related cells, underscoring its pivotal role in the acute injury response.

Further analysis of intracellular signaling flow within microglia (Fig. 2F) revealed key pathway-specific differences between Sham and 1 dpi groups. Six principal pathways were identified in the uninjured spinal cord (indicated by red bars). Highly-scoring pathways such as CCL and PTN were involved in regulating microglia activation and suppressing excessive inflammatory responses. These interactions were enhanced at 1 dpi following injury, particularly due to elevated expression of the SPP1 pathway. At 1 dpi, however, signaling flow in pathways such as SPP1 and FN1 significantly increased, suggesting that microglia became highly active and assumed a central role in orchestrating the injury response. Among these pathways, SPP1 exhibited the most pronounced upregulation, aligning with its elevated activity in global signaling patterns.

Collectively, these findings suggest that 1 dpi marks a profound reorganization of intercellular communication, with microglia emerging as central regulators of key injury-associated signaling pathways. This transition highlights the critical role of specific pathways, particularly SPP1, in orchestrating the cellular response to injury.

### CD44 in the SPP1 signaling pathway regulated the immune activation of microglia

**Fig. 3:**
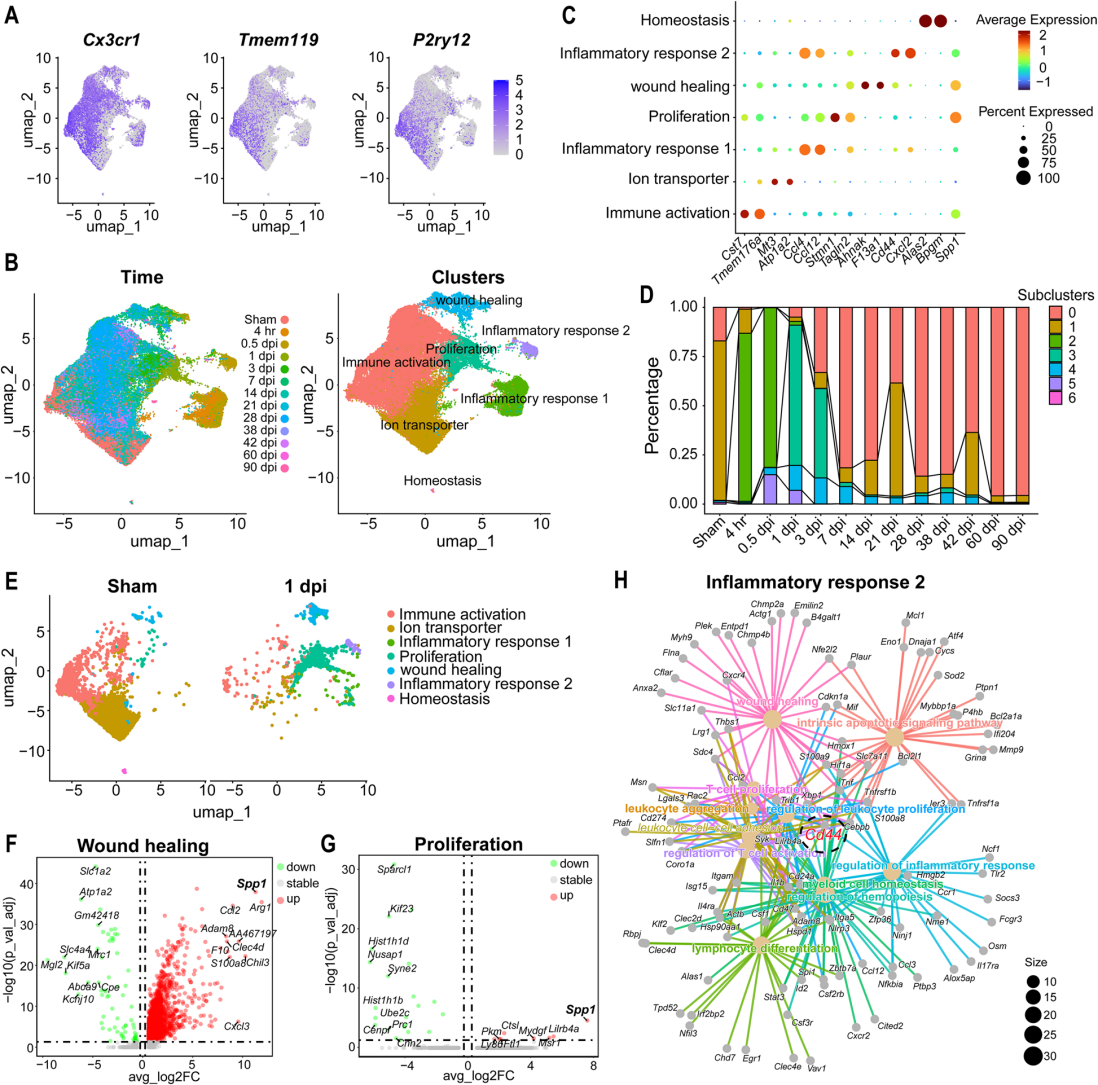
Microglial subpopulation subdivision and functional analysis showing CD44 as a key receptor regulating the inflammatory response. **A** UMAP plots illustrating the expression of microglia marker genes *Cx3cr1*, *Tmem119*, and *P2ry12* across microglia populations. **B** UMAP projections visualizing microglia from different time points post-injury, color-coded by time (left) and by cluster classification (right). Seven distinct microglial states were identified: immune activation, ion transporter inflammatory responses 1, proliferation, wound healing inflammatory responses 2, and homeostasis (corresponding to subcluster 0~6). **C** Dot plot displaying the average expression levels and percentage of cells expressing key marker genes in each microglial state. **D** Bar plot showing the proportion of cells in each subcluster across different time points. Certain states, such as immune activation and inflammatory responses, are predominant early after injury, while others, like homeostasis and ion transport, emerge later. **E** UMAP plot comparing microglial states between sham and SCI conditions at 1 dpi, showing an increase in immune activation and inflammatory responses after injury. **F**, **G** Volcano plot showing differentially expressed genes associated with the wound healing cluster and proliferation cluster. **H** Interaction network plot for the inflammatory response 2 cluster, showing the central role of *Cd44* and other key regulators in modulating inflammation post-SCI

Microglia represented the most abundant cell type in the spinal cord after SCI. Integrated analysis of all four datasets revealed distinct microglial clusters (Fig. 3A). UMAP plots of microglia marker genes *Cx3cr1*, *Tmem119*, and *P2ry12* confirmed the identity of microglial populations. These cells were further subdivided into seven functional subclusters: homeostasis, immune activation, wound healing, proliferation, ion transporter, and inflammatory responses 1 and 2, as shown in Fig. 3B and Fig. S2.

Notably, the dot plot in Fig. 3C highlights the state-specific marker genes for each microglial subcluster, while Fig. 3D illustrates the dynamic shifts in their proportions across time. In the uninjured spinal cord, Cluster 1, characterized by ion transport, was the predominant subtype. By 4 h after SCI, most microglia transitioned to the inflammatory response 1 subtype (Cluster 2), characterized by high expression of chemokine genes such as *Ccl4* and *Ccl12*. During 0.5 and 1 dpi, the inflammatory response 2 subcluster emerged, defined by elevated expression of *Cd44* and *Cxcl2*. Homeostasis markers were enriched in late injury stages, whereas immune activation and inflammatory response markers peaked early and gradually transitioned toward homeostasis and proliferation.

Comparison between sham and SCI conditions at 1 dpi (Fig. 3E) revealed significant expansion of microglial subclusters related to wound healing, proliferation, and inflammatory responses. Cell–cell communication analysis confirmed enhanced interaction among these subclusters at 1dpi (Fig. S6). Differential expression analysis of the wound healing and proliferation clusters (Fig. 3F, G) showed upregulation of key repair-related genes such as *Spp1*, *Sdc1* and *Cldn4* and proliferation markers such as *Mki67* and *Ccnd1*.

The interaction network of the inflammatory response 2 subcluster (Fig. 3H) identified *Cd44* as a central hub coordinating multiple inflammatory signals, underscoring its critical role in regulating microglial function during SCI. We noticed that inflammatory response 2 subcluster with high expression of *Cd44*, *Spp1* was also highly expressed. To characterize the inflammatory response 2 microglial subpopulation, we performed the enrichment analysis of microglial cell subpopulations features including GO and KEGG terms. Representative genes such as *Gda*, *Smox* and *Irf1* were found to be associated with regulation of innate immune response. KEGG pathway enrichment further revealed that this cluster was enriched in inflammatory pathways including the NF-κB and TNF signaling pathways (Fig. S3). These data suggest that inflammatory response 2 represents a pro-inflammatory microglial phenotype. CD44 mediated signaling may drive their inflammation activation.

### In vivo experiments confirmed the critical role of CD44 receptor in microglia activation

**Fig. 4:**
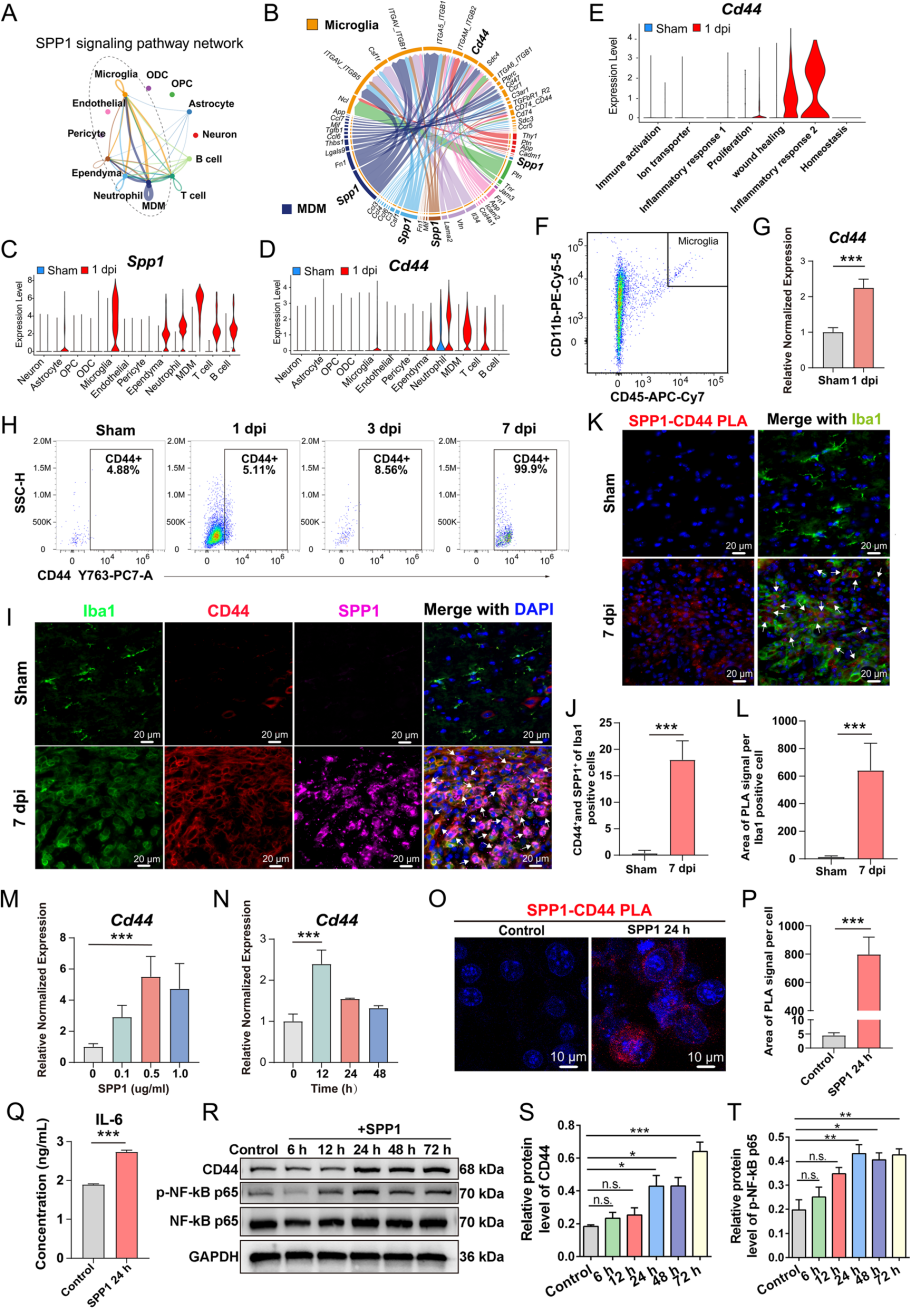
SPP1-CD44 signaling promotes microglial activation and inflammatory response. **A** SPP1 signaling pathway network showing interactions between microglia and other cell types. **B** A circular plot illustrating the interaction network of microglia with other cells in various ligand -receptor pairs, including *Spp1* -*Cd44*. **C**, **D** Violin plots showing expression levels of *Spp1* and *Cd44* across different cell types in sham (blue) and 1 dpi (red). Both genes show elevated expression in microglia following injury. **E** Violin plot of *Cd44* expression in microglia subclusters, showing the upregulation in the wound healing and inflammatory response2 cluster at 1 dpi. **F** The microglia were sorted by flow cytometry and (**G**) *Cd44* gene expression was detected by qRCR. **H** Flow cytometry analysis of CD44 positive microglia after SCI, showing a marked increase in CD44^+^ microglia during 7 dpi. **I** Immunofluorescence images of spinal cord lesion site stained for Iba1, CD44, SPP1, and merged with DAPI. White arrows indicate the co-stained CD44^+^ and SPP1^+^ signals in Iba1 positive microglia (**J**) Quantification results of CD44^+^ and SPP1^+^ in Iba1 positive microglia cells. **K** Using PLA to detect specific SPP1-CD44 interactions of spinal cord lesion site in situ. **L** Quantification of PLA results, the PLA signal is quantified and plotted as the area of PLA signal per Iba1 positive cell. **M**, **N** qRT-PCR showing dose- and time-dependent increases of *Cd44* expression in BV2 microglia after recombinant SPP1 stimulation. **O** Representative images of PLA assay specific SPP1-CD44 interactions of BV2 microglia cells in vitro. **P** PLA signal was quantified and plotted as the area of PLA signal per cell. **Q** ELISA quantification of IL-6 levels in cell supernatant after SPP1 stimulation. **R** Western blot showing the time course of CD44 and p-NF-κB p65 protein expression in BV2 cells after SPP1 treatment. (**S**–**T**) Quantification of CD44 and p-NF-κB p65 protein levels. Data are presented as mean ± SEM. (*n* = 3, *P < 0.05, **P < 0.01, ***P < 0.001)

To explore microglia-centered communication networks after SCI, we designated microglia as the central node and analyzed functionally relevant signaling pathways with the other 11 identified cell types. Using CellChat, we extracted intricate signaling patterns, particularly focusing on membrane-bound ligand-receptor interactions. Higher interaction scores reflected stronger predicted interactions between cell types through specific ligand–receptor pathways. As shown in Fig. 4A, at 1 dpi, the primary cell types interacting with microglia via the SPP1 signaling network were MDMs, ependymal cells, and neutrophils. The chord plot in Fig. 4B visualized autocrine and paracrine signaling interactions, color-coded by ligand-receptor interaction strengths. The enriched SPP1 signaling networks in microglial-MDM interactions included key receptors such as *Cd44*, *Sdc4* and integrins IGAV_ITGB5, ITGAV_ITGB1 as known contributors to SCI recovery (Bellver-Landete et al. 2019). CD44 has been implicated in regulating cell migration and the immune response at the injury sites, suggesting its potential as a therapeutic target to modulating inflammation (Luo et al. 2024).

To further investigate *Spp1* and *Cd44* expression after SCI, we analyzed their transcriptional levels across cell types at 1 dpi (Fig. 4C, D). The results showed significant upregulation of both *Spp1* and *Cd44* in microglia and other immune cells at 1 dpi. Microglia exhibited a notable increase in the expression of both genes, consistent with their activation in response to injury. Moreover, the upregulation of *Cd44* in microglial subcluster associated with wound healing and inflammatory response at 1 dpi suggests a potential role for *Cd44* in modulating inflammation and facilitating tissue repair (Fig. 4E). Enrichment analysis of inflammatory response 2 subcluster revealed associations with inflammatory pathways including the NF-κB signaling pathway (Fig. S3).

To validate the bioinformatic findings, we established a spinal cord contusion model in adult mice. Microglia isolated at 1 dpi showed significantly elevated *Cd44* mRNA levels as confirmed by qPCR (Fig. 4F, G). To further assess CD44 protein expression in microglia, microglia at the injury center were labeled and analyzed via flow cytometry. CD44^+^ microglia increased progressively from 1 to 7 dpi, with nearly all microglia expressing CD44 protein at 7 dpi (Fig. 4H, Fig. S7).

To investigate the potential interaction between SPP1 and its receptor CD44 in microglia after SCI, we performed co-immunofluorescence staining for Iba1, CD44, and SPP1. SPP1-CD44 co-localized at the lesion site, indicating enhanced ligand-receptor interactions following SCI (Fig. S4). At 7 dpi, SPP1 and CD44 were prominently co-localized in Iba1⁺ microglia, whereas minimal co-localization was observed in the sham group (Fig. 4I). Quantitative analysis revealed a significant increased proportion of Iba1 positive microglia co-localized with both SPP1 and CD44, indicating enhanced ligand-receptor interaction specifically within microglia following SCI (Fig. 4J). PLA further confirmed close spatial proximity between SPP1 and CD44 in microglia at 7 dpi, as indicated by a marked elevation in PLA signal intensity compared to controls (Fig. 4K, L).

To elucidate the functional consequence of SPP1–CD44 signaling, we treated BV2 microglial cells with recombinant SPP1, which induced a dose- and time-dependent upregulation of *Cd44* (Fig. 4M, N). PLA analysis in BV2 cells demonstrated increased SPP1–CD44 interaction after 24 h SPP1 stimulation (Fig. 4O, P). ELISA results demonstrated elevated IL-6 levels in SPP1-treated BV2 cell supernatants (Fig. 4Q), indicating a pro-inflammatory response. Furthermore, immunoblotting revealed increased CD44 protein levels and enhanced NF-κB p65 phosphorylation (Fig. 4R). Quantification results confirmed elevated CD44 and p-p65 levels at 24 h post-stimulation (Fig. 4S, T). These results indicate that SPP1 activates the NF-κB pathway to promote inflammatory responses in microglia and enhanced the expression of CD44.

These findings highlight that SPP1 plays a critical role in modulating CD44 expression in microglia. These results underscore the therapeutic potential of targeting CD44 to modulate immune response after SCI.

### Re-clustering of astrocytes indicated a key subpopulation involved in gliogenesis at 3 dpi

Following SCI, astrocytes play essential roles in responding to injury-induced stressors, including inflammation and glial scar formation (Okada et al. 2018). As the principal constituents of the glial scar encasing the lesion core, reactive astrocytes have long been considered barriers to axonal regeneration and functional recovery (Bradbury and Burnside 2019; Silver and Miller 2004). To investigate astrocyte heterogeneity and dynamic functional changes, we identified distinct astrocyte subclusters and monitored their temporal transitions post-SCI.

**Fig. 5:**
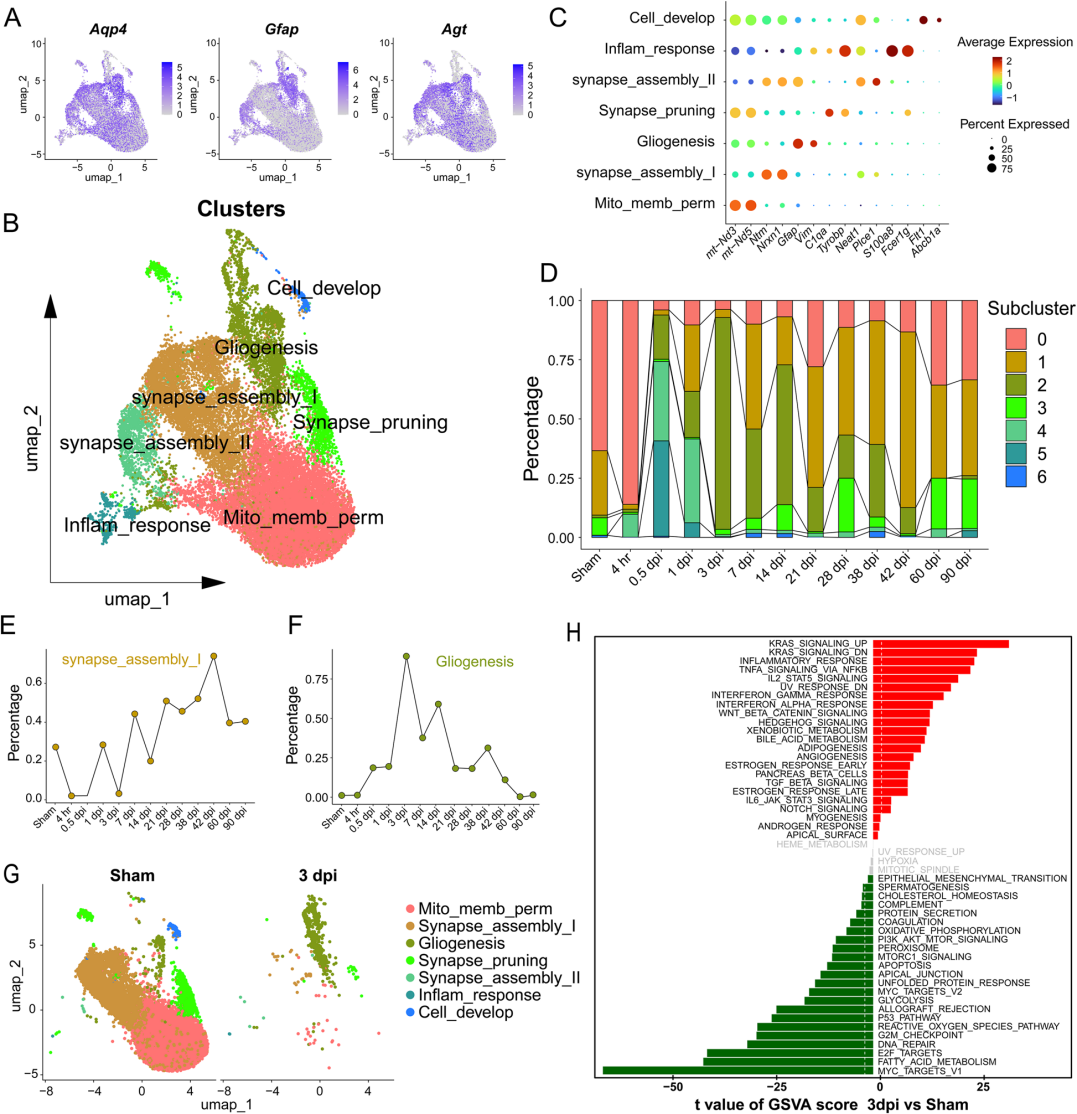
Astrocyte subcluster heterogeneity analysis and temporal dynamics indicated reactive astrocyte activation at 3 dpi. **A** UMAP plots displaying the expression of the astrocyte markers *Aqp4*, *Gfap*, and *Agt* across the astrocyte cluster. **B** UMAP plot visualizing the distinct astrocyte subpopulations involved in different biological processes, including mitochondrial membrane permeability (Mito_memb_perm), synapse assembly I, gliogenesis, synapse pruning, inflammation response (Inflam_response), synapse assembly_II, and cell development. (corresponding to cluster 0~6). **C** Dot plots showing the average expression and the percentage of cells expressing marker genes corresponding to these biological processes across astrocyte subclusters. **D** Proportional bar plot indicating the temporal dynamics of the astrocyte subclusters from sham to various time points, highlighting the peak activation of different clusters. **E**, **F** The temporal dynamics of clusters 1 and 2 indicating these subclusters respond to injury, with peaks and troughs suggesting phases of activation. **G** UMAP plots comparing the distribution of astrocyte subclusters between Sham and 3 dpi, illustrating the shift in astrocyte functions. **H** Gene Set Variation Analysis (GSVA) plot showing significantly upregulated and downregulated signaling pathways at 3 dpi compared to Sham, indicating activation of inflammatory and repair-related pathways after injury

As shown in Fig. 5A, UMAP plots displayed the expression of astrocyte markers *Aqp4*, *Gfap*, and *Agt *across astrocyte clusters. Clustering analysis revealed distinct astrocyte subpopulations engaged in critical biological processes, including cell development, gliogenesis, synapse assembly, synapse pruning, mitochondrial membrane permeability, and inflammatory responses (Fig. 5B). Figure 5C presents a dot plot of key marker genes expression associated with these biological processes across astrocyte subclusters. Temporal dynamics of the subcluster proportions were shown in Fig. 5D. Subclusters associated with mitochondrial membrane permeability, synapse assembly, and synapse pruning, (clusters 0, 1, 3) were present even in the uninjured spinal cord. Synapse assembly_II cluster (Cluster 1) was a prominent subpopulation that expanded progressively from 1 to 42 dpi. Cluster 1, characterized by* Gpc5*, *Plcb1*, *Mgat4c*, and *Lrrtm3* expression (Fig. S5), can be classified as synapse-associated neurogenic astrocytes, depending on their specific role in the brain region where they are found (de Wit and Ghosh 2016; Suh et al. 2008). These cells may support neuronal function, modulate synaptic activity, and participate in neurodevelopmental processes. By 3 dpi, the majority of astrocytes transitioned into Cluster 2, termed Gliogenesis astrocytes, marked by expression of *Vim*, *S100a6*, *Hspb1*, *Lgals1*, and *A2ms*. Particularly, *Vim* encodes vimentin, an intermediate filament critical for astrocyte reactivity (Pekny and Pekna 2014). *S100a6*, a calcium-binding protein, is involved in regulating cell proliferation, differentiation, and response to stress. In astrocytes, *S100a6* expression is associated with cell survival and repair mechanisms after injury, potentially offering a protective role during neurotrauma (Donato et al. 2013). These astrocytes exhibit a pronounced reactive phenotype following SCI, contributing to inflammatory response, cell protection, repair, and scar formation. The synapse pruning astrocytes subtype (Cluster 3), present within 12 h after SCI, express *C1qa, C1qc, Ctss1, Ly86, and Cx3cr1*, and may exhibit enhanced immune and inflammatory responses following SCI (Inoue and Tsuda 2018). These astrocytes are involved in debris clearance, regulation of the complement system (Stevens et al. 2007), and activation of immune cells (Cardona et al. 2006). These shifts highlight an early expansion of inflammatory responses and mitochondrial regulation-related astrocytes (1~7 dpi), followed by a gradual reduction as synapse assembly and gliogenesis-related subclusters became more prevalent during later stages (14~90 dpi).

Temporal changes in cluster proportions were detailed in Fig. 5E, F. Gliogenesis astrocytes (Cluster 2) showed the most significant changes after SCI, underscoring their importance in injury repair. UMAP plots comparing the distribution of astrocyte subclusters between Sham and 3 dpi illustrate a marked shift in astrocyte functional states, with an enrichment of gliogenesis astrocytes at 3 dpi (Fig. 5G).

Gene Set Variation Analysis (GSVA) corroborated these functional transitions, revealing upregulation of inflammatory pathways, including interferon signaling and IL-6 response, at 3 dpi, aligning with reactive astrocytes activation and neuroinflammatory processes (Fig. 5H). Concurrently, pathways related to metabolism and cellular homeostasis were downregulated, reflecting the cellular stress and energy demands post-injury.

Collectively, these findings highlight the critical roles of astrocytes in modulating neuroinflammation and synaptic plasticity during early SCI, suggesting that selectively targeting these specific astrocyte subpopulations may offer therapeutic benefit.

### NCL upregulated expression may mediate astrocyte activation

**Fig. 6:**
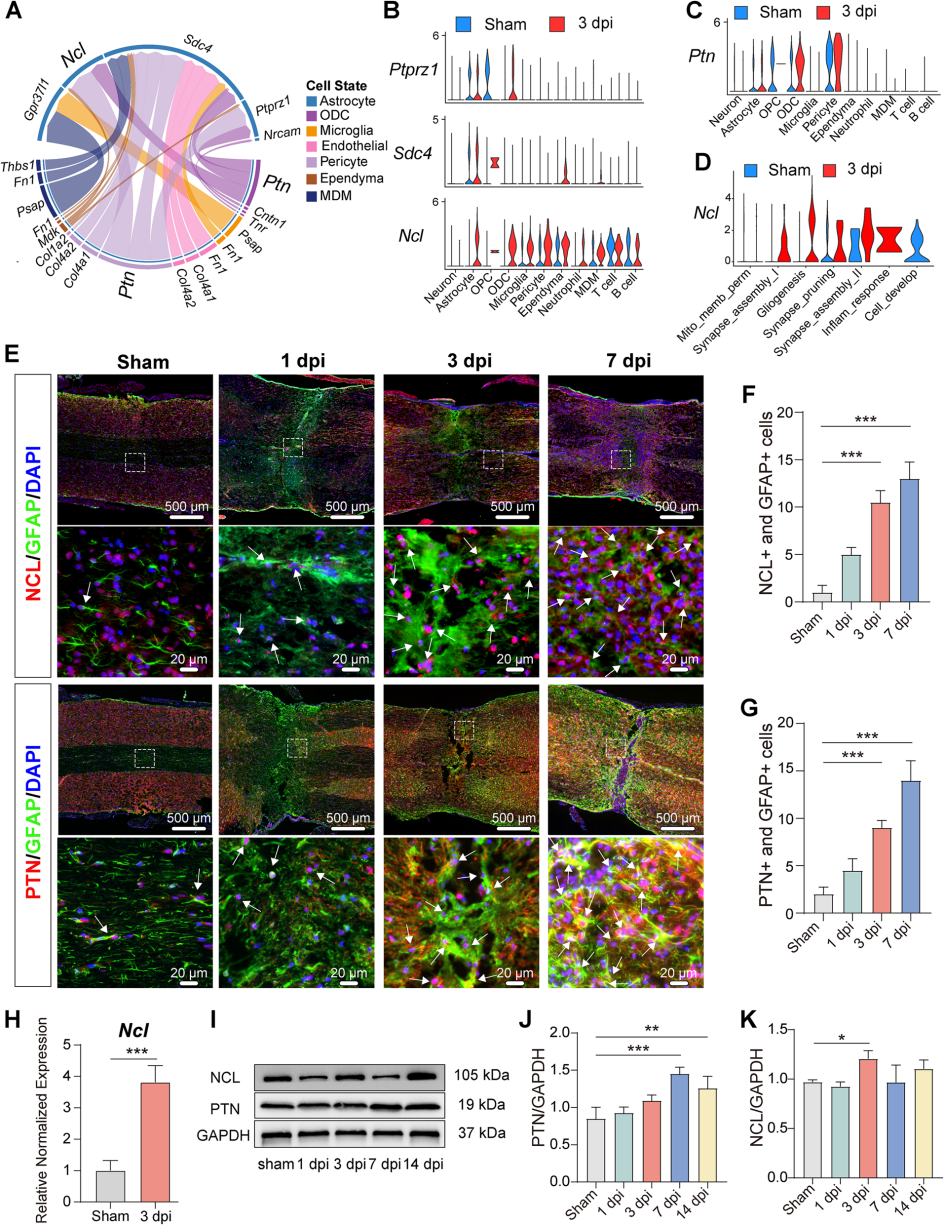
NCL upregulated expression may mediate astrocyte activation in the early response post SCI. **A** Circos plot illustrating upregulated receptor-ligand signaling from multiple cell types to astrocytes at 3 dpi, especially the *Ptn*-*Ncl* interaction. **B** Violin plots of main receptor including *Ptprz1*, *Sdc4*, and *Ncl* gene expression in different cell types at 3 dpi, with a notable increase in *Ncl* expression in astrocytes. **C** Violin plots showing the expression levels of *Ptn* across multiple cell types. **D** The expression levels of *Ncl* across astrocyte subclusters. **E** Immunofluorescence analysis showing NCL and GFAP expression, and PTN and GFAP co-localization. Arrowheads indicate positive staining. Scale bars: 500 µm (top), 20 µm (bottom). **F**, **G** Quantification of NCL^+^ GFAP^+^ cells and PTN^+^GFAP^+^ Cells showing a significant increase in NCL^+^ astrocytes at 7 dpi. **H** Quantification of relative *Ncl* gene expression in astrocytes at 3 dpi (*n* = 3). **I** Western blot analysis showing the total protein expression trends of PTN and NCL in spinal cord tissue at determined time points. **J** Quantification results of total PTN protein showed the increasing up regulation trend post SCI, and (**K**) Quantification result of NCL protein expression showing upregulation at 3 dpi, with GAPDH as control. Data are presented as mean ± SEM. (*n* = 3, *p < 0.05 **p < 0.01, ***p < 0.001)

To further investigate significantly upregulated pathways in astrocytes at 3 dpi in response to SCI, we analyzed cell–cell communication between astrocytes and other cell types. A circos plot revealed enhanced intercellular signaling directed toward astrocytes at 3 dpi (Fig. 6A) with PTN signals from multiple cell types, including ODCs and pericytes. Consistently, gene expression analysis showed a marked increase in *Ptn* expression within astrocytes, ODCs, and pericytes (Fig. 6C). The corresponding receptor analysis demonstrated that *Ncl* was highly expressed in several cell types at 3 dpi (Fig. 6B). Notably, *Ncl* expression was significantly elevated in astrocytes at 3 dpi, particularly within subclusters associated with gliogenesis and inflammatory responses (Fig. 6D), suggesting a potential role for *Ncl* in the proliferation and activation of reactive astrocytes.

Immunofluorescence staining confirmed the co-localization of NCL and PTN with GFAP^+^ reactive astrocytes, with peak expression observed at 7 dpi (Fig. 6E-G). At 3 dpi, the expression of Ncl in astrocytes was significantly increased (Fig. 6H). Western blot analysis further validated the total expression level of NCL and PTN in spinal cord tissue (Fig. 6I-K). The expression of PTN was gradually increased during 7 dpi, and NCL expression increased at 3 dpi (Fig. 6I). This was consistent with the *Ncl* gene expression (Fig. 6B), which showed elevated expression of *Ncl* at 3 dpi, potentially reflecting a widespread upregulation across multiple cell types, including astrocytes.

Together, these findings demonstrated that NCL receptor of PTN signaling is markedly upregulated in astrocytes following SCI. The temporal increase in NCL expression suggests a key role in mediating astrocyte activation and proliferation after injury.

### PTN-NCL signaling pathway promoted astrocyte proliferation after SCI

**Fig. 7:**
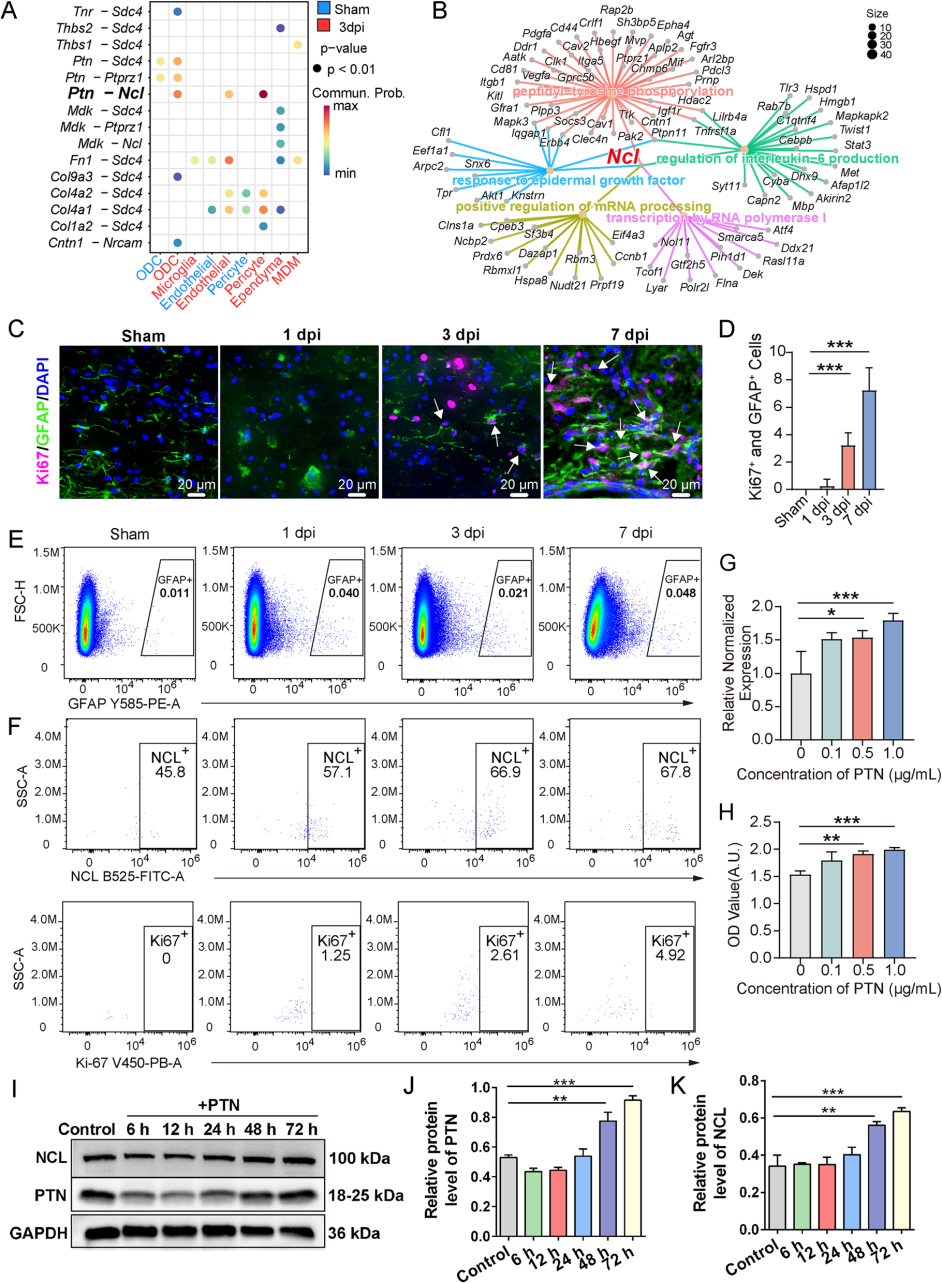
PTN–NCL signaling promotes astrocyte proliferation. **A** Dot plot showing ligand-receptor interactions between microglia, astrocytes, and other cell types with astrocytes at Sham and 3 dpi conditions. The size of the dots represents the communication probability, and the color scale indicates the significance of the interaction (*p* < 0.01). **B** Network representation of *Ptn*-mediated receptor-ligand interactions, highlighting *Ncl* as a key receptor involved in the positive regulation of epidermal growth factor signaling and interleukin-6 production. Node size represents gene expression levels, and edge width indicates interaction strength. **C** Representative immunofluorescence images showing Ki67 (magenta), GFAP (green), and DAPI (blue) in the injured spinal cord at different time points. White arrows indicate Ki67⁺ and GFAP⁺ astrocytes. **D** Quantification of Ki67⁺ and GFAP⁺ cells showing a significant increase at 7 dpi. **E** Flow cytometry analysis showing the percentage of GFAP⁺ cells in sham and SCI spinal cords. **F** Flow cytometry analysis quantifying the expression of NCL and Ki67 in astrocytes, indicates increased NCL and Ki67 expression correlating with astrocyte proliferation. **G** qRT-PCR analysis of* Ncl* expression following PTN treatment. **H** CCK-8 assay showing increased astrocyte viability after PTN stimulation. **I** Western blot showing dynamic changes in PTN and NCL protein levels at the indicated time points after PTN treatment. **J**, **K** Quantification of PTN (J) and NCL (K) protein levels, normalized to GAPDH. Data are presented as mean ± SEM. (*n*=3, *p < 0.05, **p < 0.01, ***p < 0.001)

To investigate the potential role of PTN–NCL signaling in astrocyte proliferation after SCI, cell–cell communication analysis revealed significantly enhanced ligand-receptor interactions among various cell types. The dot plot highlights increased communication probabilities between *Ptn* and several receptors such as *Ncl*, *Ptprz1*, *Ncam1*, with the highest interaction probability observed for the *Ptn-Ncl* axis (Fig. 7A). Moreover, network analysis demonstrated that *Ptn*, through its key receptor *Ncl*, contributing to positive regulation of epidermal growth factor signaling, IL-6 production, and transcription regulation and response to growth factors (Fig. 7B).

Immunofluorescence staining showed a marked increase in Ki67⁺ and GFAP⁺ astrocytes at 3 and 7 dpi (Fig. 7C), and quantification confirmed significantly elevated astrocyte proliferation at 7 dpi compared to sham (Fig. 7D). Flow cytometry further demonstrated an increase in GFAP⁺ astrocytes from 1 to 7 dpi (Fig. 7E), indicating astrocyte activation post-injury. Flow cytometry analysis revealed time-dependent changes in NCL and Ki67 expression. NCL expression also showed a marked increase, with NCL^+^ cells reaching 67.8% at 7 dpi. Additionally, Ki67^+^ cells in NCL positive astrocytes rose from 0 in the sham condition to 4.92% at 7 dpi, further confirming astrocyte proliferation after injury (Fig. 7F, Fig. S8). The percentage of NCL^+^ cells increased, and Ki67^+^ cells also significantly increased at 3 dpi and 7 dpi compared to Sham, indicating that NCL receptor may facilitate cell proliferation following injury.

As shown in Fig. 7G, a dose-dependent increase in *Ncl* expression was observed. CCK8 assay revealed that PTN treatment significantly increased cell proliferation at 1 µg/mL, indicating that PTN promotes astrocytes proliferation (Fig. 7H). To assess the protein level of NCL after PTN treatment, we performed in vitro experiments using the mouse astrocyte cell line C8-D1A. Cells were treated with recombinant mouse PTN (1 μg/mL). Astrocytes cultured for 72 h without PTN treatment served as control group. The PLA results revealed red dot fluorescent signals indicating PTN-NCL binding in astrocytes at 24 h with PTN treatment. We also observed the PLA signal within the nucleus, as shown by the white circles (Fig. S9). These data suggest that exogenous PTN may bind to surface NCL in astrocytes and promote nuclear localization. NCL protein levels were increased at 48 h post-treatment (Fig. 7K), suggesting a response to PTN stimulation. Densitometric quantification showed a time-dependent elevation of both PTN and NCL (Fig. 7J, K), suggesting a potential positive feedback loop. Collectively, these findings suggest that PTN–NCL interaction contributes to astrocyte proliferation in response to SCI.

## Disscussion

In this study, we identified CD44 and NCL as key receptors in the cellular responses following SCI, providing new insights into the mechanisms of inflammation and gliogenesis. Here, we discuss the potential mechanisms underlying these functional changes and their implications for therapeutic strategies.

One important receptor for SPP1 is CD44, a widely expressed multifunctional transmembrane glycoprotein involved in immune cells activation (Dzwonek and Wilczynski 2015; Marcondes et al. 2008). We identified SPP1-CD44 pairs that regulated the microglia activation. Specifically, MDMs, in direct contact with microglia, exhibited the highest level of communication in the SPP1 signaling pathway. We anticipated that microglia predominantly receive signals from MDMs and trigger immune activation from their original states. The variation in the crush injury model demonstrated the co-expression of SPP1-CD44 pairs in microglia and enhanced microglial activation at the injury site. The proliferating microglial cells formed typical glial boundaries (Bellver-Landete et al. 2019), which can promote the adhesion of MDMs and limit the range of inflammatory reactions in the damaged area, thereby protecting normal spinal cord tissue. Furthermore, CD44 is a critical regulator of cellular migration and cell–cell matrix interactions in many cell types (Dzwonek and Wilczynski 2015; Morath et al. 2016). Many immune cells present post-SCI are not native to the spinal cord and thus have to infiltrate into the spinal cord via the periphery. It was demonstrated that CD44 knockout mice exhibit fewer immune cells at the injury site compared to wild-type mice (Creasman 2023), indicating that immune cells rely heavily on CD44 to reach their target, and thus more likely to be affected by its absence. This evidence may illustrate the results presented in this paper that microglia regulate immune cell activation. We found that CD44 expression increased significantly in microglia within 7 dpi. This finding is consistent with previous studies showing that CD44 plays a pivotal role in immune cell recruitment and inflammatory responses (Yim et al. 2022). In this study, SPP1 treatment promoted phosphorylation of NF-κB p65 indicating that the expression of CD44 may occur through the NF-κB pathway. This is consistent with previous reports that upregulation of *Cd44* transcription via NF-κB pathway activation (Smith et al. 2014). The activation of CD44 by its ligand SPP1 likely triggers a series of intracellular signaling cascades that enhance microglial activation. Notably, microglia become more motile and adopt a pro-inflammatory phenotype, secreting cytokines such as IL-6, as confirmed in our ELISA results, which contribute to the neuroinflammatory environment following SCI. These cytokines exacerbate tissue damage and hinder neuronal repair. This suggests that CD44 could be a potential therapeutic target for modulating microglial activation and controlling excessive inflammation after SCI.

Reactive astrogliosis following SCI causes astrocytes to proliferate and become the predominant cellular component of the glial scar (Clifford et al. 2023). Traditionally, the astrocytic scar has been viewed as a barrier that inhibits axonal regrowth due to the deposition of inhibitory extracellular matrix components such as CSPGs (Silver and Miller, 2004). However, recent studies have highlighted the positive role of astrocytic scars that in limiting inflammation, preventing further tissue damage, and maintaining structural stability of the injured area (Anderson et al. 2016). Therefore, targeting astrocytes or scar modulation must be approached with caution to avoid disrupting these beneficial effects. Developing therapeutic strategies that selectively target specific astrocyte subpopulations during astrogliosis is crucial. In this study, we found that a distinct population gliogenesis subcluster at 3 dpi that expressed high levels of *Ncl*, a protein primarily involved in RNA processing and transcriptional regulation. NCL is a multifunctional protein that can exist both in the nucleus of cells and on the surface, involved in the maturation of ribosomes (Lamprou et al. 2022). Previous research has revealed that NCL interacts with EGFR to mediate astrogliosis after SCI, and inhibition of NCL by the aptamer GroA (AS1411) effectively disrupts this NCL-EGFR interaction, results in attenuation of astrocyte reactivity and proliferation in *vivo* and in vitro (Goldshmit et al. 2016). PTN-NCL interaction is involved in neuroregeneration and tissue repair by activating signaling pathways that influence cell proliferation (Landgraf et al. 2008; Sofroniew 2015). It has been demonstrated that elevated PTN levels enhanced neurofibroma-associated fibroblasts proliferation and collagen synthesis through NCL receptor (Tian et al. 2024). As shown in Fig. 7I-K, NCL protein levels were increased at 48 h post-treatment, suggesting a response to PTN stimulation. Importantly, we also observed increased PTN protein levels at 72 h after PTN treatment compared with control (Fig. 7J). We supposed that there might be two possible reasons: (1) astrocytes endogenously secrete a certain amount of PTN via signaling (Linnerbauer et al. 2022), and (2) astrocytes are capable of internalizing exogenous PTN following external stimulation. This may illustrate the significantly elevated total PTN levels in astrocytes at 72 h after PTN treatment compared to the untreated controls. The upregulation of its receptor NCL, which may further enhance PTN–NCL interaction and internalization. This may be supported by the previous studies showing that PTN binds to cell-surface NCL, leading to its internalization and nuclear translocation (Sorrelle et al. 2017). In the current research, the precise mechanism of PTN–NCL signaling still remains unclear and requires further clarification.

Glial scar formation is an essential part of the early-stage repair process, as it protects neurons from further damage and prevents excessive inflammation. However, persistent scar tissue can inhibit axonal regeneration and recovery. The precise regulation of NCL receptor may therefore be critical in balancing scar formation and neural regeneration.

Despite the promising findings presented in this paper, future validation and functional characterization of CD44 and NCL receptors on microglia and astrocyte are necessary. Conditional knockdown or overexpression of CD44 and NCL is needed to clarify the effect on the function of SCI.

## Conclusions

In this study, a major innovation of this study is the identification of CD44 as a pivotal receptor in the SPP1-mediated microglial activation pathway, a critical mechanism that drives the inflammatory response immediately following SCI. This novel finding not only enhances our understanding of microglial dynamics in the acute phase of injury but also provided CD44 as a potential therapeutic target for modulating inflammatory response post-SCI. Moreover, we identified a gliogenic astrocyte subpopulation at 3 dpi, driven by the activation of NCL through the PTN signaling axis. This discovery highlights the previously underexplored role of NCL in astrocyte proliferation during SCI recovery. Our results provide new insights into potential therapeutic targets for SCI.

## Methods

### Data source and processing

The mouse SCI data of scRNA-seq were collected from several samples and downloaded from the NCBI GEO (Barrett et al. 2013) (GSE189070, GSE196928, GSE172167) and Figshare (Singh. 2011) repository (17702045). For GSE196928, we removed the samples with PLX5622 treatment to keep the phenotype of all results only effected by the SCI. The data of all scRNA-seq samples were further processed with the unified format of cell type and developmental time point.

### Integration analysis of scRNA-seq data

To make all scRNA-seq samples analyzable without any batch effects, the data matrices of these samples were integrated using the reciprocal principal component analysis (RPCA) method by the Standard Seurat v 5.0.3 Integration Workflow. Before integration, quality control was applied to each data matrix, which involved the removal of genes detected in no more than 3 cells and cells with up to 200 detected genes or less than 20% of reads coming from mitochondrial. Additionally, for each data matrix, the top 2,000 highly variable genes were selected for the next principal component analysis (PCA), of which the number of principal components was set as 50 to ensure extraction of enough features from the matrix.

### Clustering and re-clustering with cell type annotation

Clustering was performed in two phases on (1) all cell types, (2) specifically within microglia and astrocytes. For phase 1, the top 2,000 highly variable genes were detected using the “FindVariableFeatures” function, and 30 PCs were used for clustering. For phase 2, raw data from all cells in microglia and astrocytes were re-scaled, re-normalized, and re-integrated respectively. The top 4,000 highly variable genes were detected and the top 40 PCs were selected for re-clustering.

In phase 1, we used the following method to determine the cell type: keep the cell type annotation information in meta.data of rds files. For clusters without annotation, the function “FindAllMarkers” was used to find the differentially expressed genes (DEGs) in each cluster. Significantly differentially expressed genes had an adjusted p value of less than 0.01 and the cutoff of log_2_-fold change ‘logfc.threshold’ was set as 0.75. The cell type of each cluster was identified by using several marker genes reported in the literature (Neuron: *Snap25, Rbfox1, Rbfox3*; Astrocyte: *Aqp4, Gfap, Agt*; OPC: *Pdgfra, Tnr, Cspg5*; ODC: *Plp1,Mag, Mog*; Microglia: *Cx3cr1, Tmem119, P2ry12*; Endothelial: *Cldn5, Pecam1, Ly6c1*; Pericyte: *Pdgfrb, Vtn, Myl9*; Ependyma: *Foxj1, Nnat, Rsph1*; Intermediate progenitors: *Stmn1*, *Hmgb2*, *Top2a*; Neutrophil: *Ly6g*, *S100a8*, *S100a9*; Monocyte: *Ly6c2*, *Ccr2*; MDMs: *Gpnmb*, *Spp1*, *Fabp5*; T cells: *Ccl5*, *Ms4a4b*, *Nkg7*; B cells: *Ly6d*, *Cd79a*, *Cd79b*; Leukocyte: *Ltb*, *Ctsw*, *Cd3e*; Erythrocyte: *Hba*-*a2*, *Hbb*-*bs*, *Alas2*; Stromal: *Dcn*, *Apod*, *Col1a1*, *Col3a1*; Leptomeninges: *Mgp*, *Igfbp6*, *Gsn*; Schwann: *Mpz*, *Pmp22*) (Brennan et al. 2022; Hou et al. 2022; Li et al. 2022; Matson et al. 2022; Milich et al. 2021a). UMAP location were used to systemically identify closely related clusters.

### Differential expression testing and Function Enrichment Analysis

To identify differentially expressed genes, we used the “FindMarkers” function in Seurat and compared gene expression of cells within 3 dpi, 1 dpi versus Sham in microglia and astrocytes, respectively. Significantly differentially expressed genes had an adjusted p value of less than 0.01 and the Seurat command ‘logfc.threshold’ was 0.75. Function enrichment analysis of Gene Ontology (GO) was performed by enrichGO function in R package cluster Profiler v 4.11.0 and DOSE v 3.24.2 (Wu et al. 2021). GO biological process, cell compartment and molecular function were analyzed. Kyoto Encyclopedia of Genes and Genomes (KEGG) was performed by gseKEGG function. Adjusted p value less than 0.05 were used as significant cutoff.

### Cell–cell communication analysis

Cell–cell communication was conducted using R package CellChat v1.6.1 (Jin et al. 2021a). We isolated the astrocyte, endothelial, ependyma, microglia, neuron, ODC, OPC, pericyte, B cells, T cells, MDMs and neutrophil within each of three conditions, Sham, 3 dpi and 7 dpi. We then created the CellChat object for each cell type of the three experimental conditions using the “createCellChat” function. The comparison of cell communication among different timepoints included sub-clusters with more than 50 cells in each sample. The ligand–receptor interactions used in analysis come from the literature-supported “CellChatDB.mouse” database of CellChat. The identification of major signals for specific cell groups and global communication patterns were based on an unsupervised learning method “non- negative matrix factorization”. The “identifyOverExpressedGenes” and “identifyoverExpressedfInteractions” functions were utilized to identify the overexpressed genes and ligand-receptor interactions. The cell–cell communication probability was calculated by the “computeCommunProb” functions. The cell–cell communications among different cell types within each experimental condition were visualized using chord, scatter and bubble diagrams.

### In vivo animal experiments

#### Animals

All experimental procedures using laboratory animals were conducted in accordance with the Guide for the Care and Use of Laboratory Animals (National Research Council, National 1996) and approved by the Animal Care and Use Committee of Shenzhen Top Biotech Co. Ltd (No. TOP-IACUC-2023–0219). Female C57BL/6 mice (Eight-week-old, weighing 18 ~ 20 g) were purchased from the Guangdong Medical Laboratory Animal Center and housed under standard specific pathogen free (SPF) conditions. All animals were housed in standard cages under a regulated environment (12 h light/dark cycle) with free access to food and water about 1 week before surgery.

#### Adult crush injury model surgeries

Adult mice underwent T10 crush injury as previously described (Li et al. 2022; Li et al. 2020). Briefly, a midline incision was made over the thoracic vertebrae, followed by a laminectomy at T9–T11. The tips of forceps were carefully inserted on either side of the spinal cord to encompass its full width, ensuring that no tissue was spared ventrally or laterally. The spinal cord was then fully crushed for 3 s using 0.1-mm forceps (Video S1). After the injury, the muscles were sutured, and the skin was closed with sutures. Mice were placed on a warming pad post-surgery until fully awake and were administered artificial urination (twice daily for 14 dpi surgery). At 14 dpi, hind limb movements were recorded to demonstrate motor dysfunction induced by spinal cord contusion. (Video S2).

#### Spinal cord section

Animals were euthanized using a lethal dose of 4% Nembutal, and then transcardially perfused with normal saline and 4% paraformaldehyde (PFA, Biosharp, Cat No. BL539A). The spinal cord tissue about 1 cm with the lesion core in the center was removed and fixed in 4% PFA overnight, and then incubated in 20% sucrose in PBS for 24 h, followed by incubation in 30% sucrose for another 24 h at 4 °C. The tissues were then embedded in tissue embedding compound (OCT, SAKURA, Cat No. 4583), frozen, and stored at − 80 °C. Transverse and sagittal sections were cut at 10 µm thickness using a cryostat and stored at − 20 °C.

#### Total protein extraction of spinal cord

Three randomly selected animals were used at predetermined time points, including sham, 1 dpi, 3 dpi, 7 dpi, and 14 dpi. Animals were transcardially perfused with normal saline. A segment of the spinal cord, 3 mm rostral and caudal to the injury site, was dissected. Total protein was extracted by homogenizing the tissue in a lysis buffer, followed by centrifugation at 12,000 g for 10 min at 4 °C and quantitated by a BCA assay.

### Immunofluorescent staining

Before staining, sections were warmed to room temperature for 1 h, treated with 0.3% Triton X-100 for 15 min, and blocked with 5% BSA for 30 min at room temperature. The primary antibodies used included: mouse anti-GFAP (CST, Cat No. 3670S, 1:200); rabbit anti-NCL (CST, Cat No. 14574S, 1:200); rabbit anti-PTN (Bioswamp, Cat No. PAB59860, 1:100); mouse anti-CD44 (Affinity, Cat No. BF9213, 1:100); rabbit anti-SPP1 (Bioswamp, Cat No. PAB52737, 1:100); goat anti-Iba1 (Abcam, Cat No. ab289874 1:1000) and rabbit anti-Ki67 (Abcam, Cat No. ab15580, 1:1000). The secondary antibodies included Alexa Fluor 488-conjugated goat anti-mouse (Invitrogen, Cat No. A-10680, 1:500), Alexa Fluor 488-conjugated donkey anti-goat (Invitrogen, Cat No. A32814, 1:500), Alexa Fluor 568-conjugated goat anti-mouse (Abcam, Cat No. Ab175473, 1:500), and Alexa Fluor 647-conjugated goat anti-rabbit antibodies (Invitrogen, Cat No. A-21244, 1:500). Spinal cord sections were imaged using the Olympus VS 200 scanning system.

### Flow cytometry analysis

#### Tissue dissociation

A total of three animals were used for each of the two biological replicates. A 3 mm section of the spinal cord, centered at the injury site, was carefully dissected. The tissue was finely chopped into approximately 1 mm pieces using a razor blade, washed with 5 ml of PBS, and centrifuged at 300 g for 5 min at 4 °C. The resulting cell pellet was processed using the Spinal cord tissue dissociation kit (AccuraMed, Cat No. AM-SJ2403053C) according to the manufacturer’s instructions to obtain a single-cell suspension. Briefly, the pellets were incubated in 2 ml of Enzyme Mix 1 for 25 min at 37 °C, with gentle agitation by hand every 5 min. The suspensions were then strained through a 100 µm cell strainer, followed by a 40 µm strainer. After centrifugation at 500 g for 5 min at 4 °C, the supernatants were discarded. The cell pellets were resuspended in 3 ml of PBS, followed by the addition of a defragmentation reagent and gentle mixing. Next, 4 ml of PBS was carefully added along the tube wall without mixing to form layers. The mixture was centrifuged at 1200 g for 12 min at 4 °C. The cell pellet was resuspended in 10 ml of PBS, washed with 10 ml of DPBS at 4 °C for 5 min at 300 g, and finally resuspended in 500 µl of DPBS. The cells were counted, and the concentration was adjusted to approximately 10^6^ cells/mL.

#### Flow cytometry

The cell pellet was then resuspended in 100 µl of 1% BSA buffer. To identify live cells, the pellet was incubated with Fixable Viability Stain 510 (BD Pharmingen, Cat No. 564406, 1:100) for 30 min at room temperature, followed by washing with PBS and centrifugation at 300 g.

For the identification of the CD44 receptor on microglia, cells were resuspended in 100 µl of antibody mixture buffer containing 1 µl APC-Cy7 rat anti-mouse CD45 (BD Pharmingen, Cat No. 561037), 1 µl PerCP-Cy5.5 rat anti- mouse CD11b (BD Pharmingen, Cat No. 561114), and 1 µl PE-Cy7 rat anti-mouse CD44 (BD Pharmingen, Cat No. 560569). After incubation, cells were washed and resuspended in 1 ml of 4% FBS, then analyzed using a CytoFLEX Flow Cytometer (Beckman Coulter).

For the identification of the NCL receptor on astrocytes, cells were first stained for viability and then washed with 1 ml of Perm/Wash solution, followed by incubation at room temperature for 40 min. After washing twice with Perm/Wash buffer and centrifugation at 300 g for 5 min, the cells were incubated in a blocking buffer mixture containing 3 µl NCL rabbit mAb (CST, Cat No. 14574) and 99 µl PBS for 45 min at 37 °C. Subsequently, the cells were washed twice with 1 ml of PBS, centrifuged at 300 g for 5 min, and then incubated with an antibody mixture containing PE mouse anti-GFAP (BD Pharmingen, Cat No. 561483), BV421 mouse Anti-Ki-67 (BD Pharmingen, Cat No. 562899), and goat anti-rabbit Alexa Fluor 488 conjugate (Invitrogen, Cat No. A-11008, 1:500). After incubation, the cells were washed and resuspended in 1 ml of 4% FBS, then analyzed using a CytoFLEX Flow Cytometer (Beckman Coulter).

#### Flow sorting of microglia and astrocytes

Animals of sham, 1 dpi and 3 dpi were digested into single cell suspensions as descript above. There were 6 animals in each group to collect adequate number of cells. We used 1 µl of APC-Cy7 rat anti-mouse CD45 (BD Pharmingen, Cat No. 561037) and 1 µl of PerCP-Cy5.5 rat anti- mouse CD11b (BD Pharmingen, Cat No. 561114) to label microglia, and PE-Vio770 anti-mouse ACSA-2 (Miltenyi, Cat No. 130–116-246) to label astrocytes. The BD FACS Aria TM III was used for sorting. The sorted microglia and astrocytes were then lysis for quantitative reverse transcription polymerase chain reaction (qRT—PCR) experiments.

### In vitro experiments

#### Cell culture

BV2 microglial cells (Procell, Cat No. CL-0493) and C8-D1A (Procell, Cat No. CL-0506) astrocytes were seeded in 6 well plates at a density of 2 × 10^5^ cells per well. After adhesion, recombinant mouse SPP1 protein (MCE, Cat No. HY-P71786) and recombinant mouse PTN protein (MCE, Cat No. HY-P71213) were separately administered to the microglia and astrocytes at concentrations of 0, 0.1, 0.5, and 1 µg/mL for a duration of 24 h. Subsequent to the stimulation period, the culture medium was carefully removed, and the cells were gently washed twice with PBS. Total RNA was then extracted, and the expression levels of *Cd44* in microglia and *Ncl* in astrocytes were quantitatively measured using quantitative reverse transcription polymerase chain reaction (qRT—PCR). Once the effective concentration of SPP1 was determined, BV2 cells were treated with 0.5 µg/mL of recombinant SPP1 protein for 12, 24, and 48 h. Total RNA was extracted at each specific time point, and qRT-PCR was carried out to evaluate the expression of the *Cd44* gene. Simultaneously, the CCK8 assay was employed to assess the proliferation capacity of astrocytes.

#### Total protein extraction of cells

BV2 cells were treated with 0.5 μg/mL SPP1 for 6, 12, 24, 48, and 72 h. Similarly, C8-D1A astrocytes were treated with 1 μg/mL PTN at the same time points. At determined points, cells were washed with cold PBS and lysed in RIPA buffer containing 1% protease and phosphatase inhibitors.

#### mRNA expression analysis

The mRNA expression levels of *Cd44* (forward primer: 5’ -TCGATTTGAATGTAA CCTGCCG-3’; reverse primer: 5’-CAGTCCGGGAGATACTGTAGC-3’) and *Ncl* (forward primer: 5’-AAAGGCAAAAAGGCTACCACA-3’; reverse primer: 5’-GGAATGACTTTGGCTGGTGTAA-3’) were quantified using quantitative reverse transcription polymerase chain reaction, with *Gapdh* (forward primer: 5’-AGGTCGGTGTGAACGGATTTG-3’; reverse primer: 5’-GGGGTCGTTGATG GCAACA-3’) serving as the internal control. Total RNA was isolated from microglia and astrocytes and reverse-transcribed following the manufacturer’s instructions. qRT-PCR was performed on the cDNA of each sample and target gene using Taq Pro Universal SYBR qPCR Master Mix to assess the signal intensity. The relative fold changes in mRNA expression were calculated using the 2^−ΔΔCt^ method.

#### Western blot analysis

Samples were loaded onto a 10% polyacrylamide gel and separated by SDS–PAGE. The proteins were then transferred to polyvinylidene fluoride (PVDF) membranes and blocked in 5% skimmed milk in PBST for 1 h at room temperature. The membranes were incubated overnight at 4 °C with primary antibodies, including mouse anti-CD44 (Affinity, Cat No. BF9213, 1:1000), rabbit anti-p65 (Servicebio, Cat No. GB11997), rabbit anti-phospho-p65 (Servicebio, Cat No. GB113882), rabbit anti-NCL (Proteintech, Cat No. 10556–1-AP,1:1000), rabbit anti-PTN (Bioswamp, Cat No. PAB59860, 1:1000), and rabbit anti-GAPDH (Proteintech, Cat No. 10494–1-AP, 1:5000). After washing three times with PBST, the membranes were incubated with HRP-conjugated mouse secondary antibodies (Proteintech, Cat No. SA00001-1, 1:5000) or HRP-conjugated rabbit secondary antibodies (Proteintech, Cat No. SA00001-2, 1:5000). Protein bands were visualized using an ECL kit (Affinibody, Cat No. AIWB-006) and quantified using the Image Lab Software (Bio-Rad). and band intensities were quantified using ImageJ software.

#### Enzyme-linked immunosorbent assay (ELISA) for IL-6 detection

Cell culture media of BV2 microglial cells treated with or without SPP1 for 48 h were harvested, centrifuged at 3,000 × g for 10 min to eliminate cellular debris. The levels of IL-6 in the collected supernatants were assessed using the mouse IL-6 ELISA kit (Invitrogen, Cat No. ECM007) following the manufacturer's protocol. The optical density was recorded at 450 nm using a microplate reader. IL-6 concentrations were calculated from a standard curve.

#### Proximity ligation assay (PLA)

Ligand-receptor interactions between CD44–SPP1 and NCL–PTN were detected using the Duolink® in situ red kit (Sigma-Aldrich, DUO92102). BV2 microglia or C8-D1A astrocytes were seeded on poly-L-lysine-coated coverslips and treated with 0.5 μg/mL recombinant SPP1 or 1 μg/mL PTN for 24 h. Cells were fixed in ice methanol for 10 min, permeabilized with 0.1% Triton X-100, and blocked for 1 h at 37 °C. Primary antibodies: rabbit anti-NCL (CST, Cat No. 14574S, 1:200); mouse anti-PTN (Santa Cruz, Cat No. sc-74443, 1:25); mouse anti-CD44 (Affinity, Cat No. BF9213, 1:100); rabbit anti-SPP1 (Bioswamp, Cat No. PAB52737, 1:100); goat anti-Iba1 (Abcam, Cat No. ab289874 1:1000) were incubated overnight at 4 °C. Species-specific PLA probes were applied, followed by ligation and amplification per the manufacturer’s instructions. Spinal cord sections of sham and 7 dpi were detected with this method. Goat anti-Iba1 antibody was incubated together with the PLA probe. After the stain process of PLA, Alexa Fluor 488-conjugated donkey anti-goat (Invitrogen, Cat No. A32814, 1:500) was incubated for 1 h. Nuclei were stained with DAPI, and red fluorescent PLA signals were visualized by confocal microscopy. Quantification as determined by area of PLA signal per field, or by area of PLA signal per field/number of cells using ImageJ (Hegazy et al. 2020).

#### Data availability

The publicly available data utilized in this study are available at:

https://www.ncbi.nlm.nih.gov/geo/query/acc.cgi?acc=GSE189070 (Hou et al. 2022).

https://www.ncbi.nlm.nih.gov/geo/query/acc.cgi?acc=GSE196928 (Brennan et al. 2022) 

https://www.ncbi.nlm.nih.gov/geo/query/acc.cgi?acc=GSE172167 (Matson et al. 2022).

https://doi.org/10.6084/m9.figshare.17702045 (Li et al. 2022).

#### Statistical analysis

All experiment were performed in triplicate technical replicates, and data are presented as means ± standard deviations (SD). For bioinformatics analysis, tests involving comparisons among multi-groups were performed using Kruskal–Wallis rank-sum test or ANOVA, and comparisons between two-groups were performed using Wilcoxon rank-sum test or t-test. And the result with p value < 0.05 was considered to be statistically significant.

## Supplementary Information

Supplementary Material 1: Fig. S1~S9. Fig. S1: Comparative analysis of single-cell RNA sequencing datasets from different studies. Fig. S2: Microglia subpopulation subdivision and functional analysis. Fig. S3: The enrichment analysis of microglia subcluster features includes GO and KEGG terms. Fig. S4: Spatial co-localization of SPP1 and CD44 in spinal cord sections after SCI. Fig. S5: Astrocyte subpopulation subdivision and functional analysis. Fig S6: Analysis of cell communication between microglia subsets and astroglia subsets 7 days after injury. Fig. S7: Flow cytometry results were used to identify live cells and CD44-positive microglia. Fig. S8: Flow cytometry results were used to determine the results of live cells, NCL, and Ki67 positive astrocytes. Fig. S9: PTN interaction with NCL in astrocytes as detected by PLA.

Supplementary Material 2: Table S1: A detailed description of the animal model in the four databases used in the integrated analysis.

Supplementary Material 3: Video S1: Record of the modeling process of spinal cord injury crush model.

Supplementary Material 4: Video S2: Hind limb movement of mice at 14 dpi.

## Data Availability

Data in this paper are openly available.

## Abbreviations

SCI: Spinal Cord Injury
dpi: Days Post Injury
scRNA-seq: Single-Cell RNA Sequencing
snRNA-seq: Single-neucle RNA Sequencing
MDMs: Monocyte-Derived Macrophages
Ncl: Nucleolin
Ptn: Pleiotrophin
GFAP: Glial Fibrillary Acidic Protein
Ki67: A protein associated with cell proliferation
BSA: Bovine Serum Albumin
PBS: Phosphate-Buffered Saline
FBS: Fetal Bovine Serum
CCK-8: Cell Counting Kit-8
qRT-PCR: Quantitative Reverse Transcription Polymerase Chain Reaction
GO: Gene Ontology
KEGG: Kyoto Encyclopedia of Genes and Genomes
RNA-seq: RNA Sequencing
PCA: Principal Component Analysis
UMAP: Uniform Manifold Approximation and Projection
FACS: Fluorescence-Activated Cell Sorting
ELISA: Enzyme-Linked Immunosorbent Assay
PLA: Proximity Ligation Assay
CSPG: Chondroitin Sulphate Proteoglycans
