# Supplementary material for: Integrative analysis and experimental validation identify the role of CD44 and Nucleolin in regulating gliogenesis following spinal cord injury
Source: Cell Regen. 2025 Aug 13;14:35. doi: 10.1186/s13619-025-00253-x (PMC12344060; doi:10.1186/s13619-025-00253-x)
Supplement: Supplementary file 1 — Supplementary Material 1: Fig. S1~S9. Fig. S1: Comparative analysis of single-cell RNA sequencing datasets from different studies. Fig. S2: Microglia subpopulation subdivision and functional analysis. Fig. S3: The enrichment analysis of microglia subcluster features includes GO and KEGG terms. Fig. S4: Spatial co-localization of SPP1 and CD44 in spinal cord sections after SCI. Fig. S5: Astrocyte subpopulation subdivision and functional analysis. Fig S6: Analysis of cell communication between microglia subsets and astroglia subsets 7 days after injury. Fig. S7: Flow cytometry results were used to identify live cells and CD44-positive microglia. Fig. S8: Flow cytometry results were used to determine the results of live cells, NCL, and Ki67 positive astrocytes. Fig. S9: PTN interaction with NCL in astrocytes as detected by PLA. [file 13619_2025_253_MOESM1_ESM.docx]

**Supplemental information for**

**Integrative analysis and experimental validation identify the role of CD44 and Nucleolin in regulating gliogenesis following spinal cord injury**

Ming Shi^1, #^, Yazhou Sun^1, 3, #^, Lu Ding^1^, Xinyue Li^1^, Qi Xu^1^, Fuxin Wei^2, 4,^ *, Tianshun Gao^1, 3, 4^ *, David Y.B. Deng^1, 4,^ *


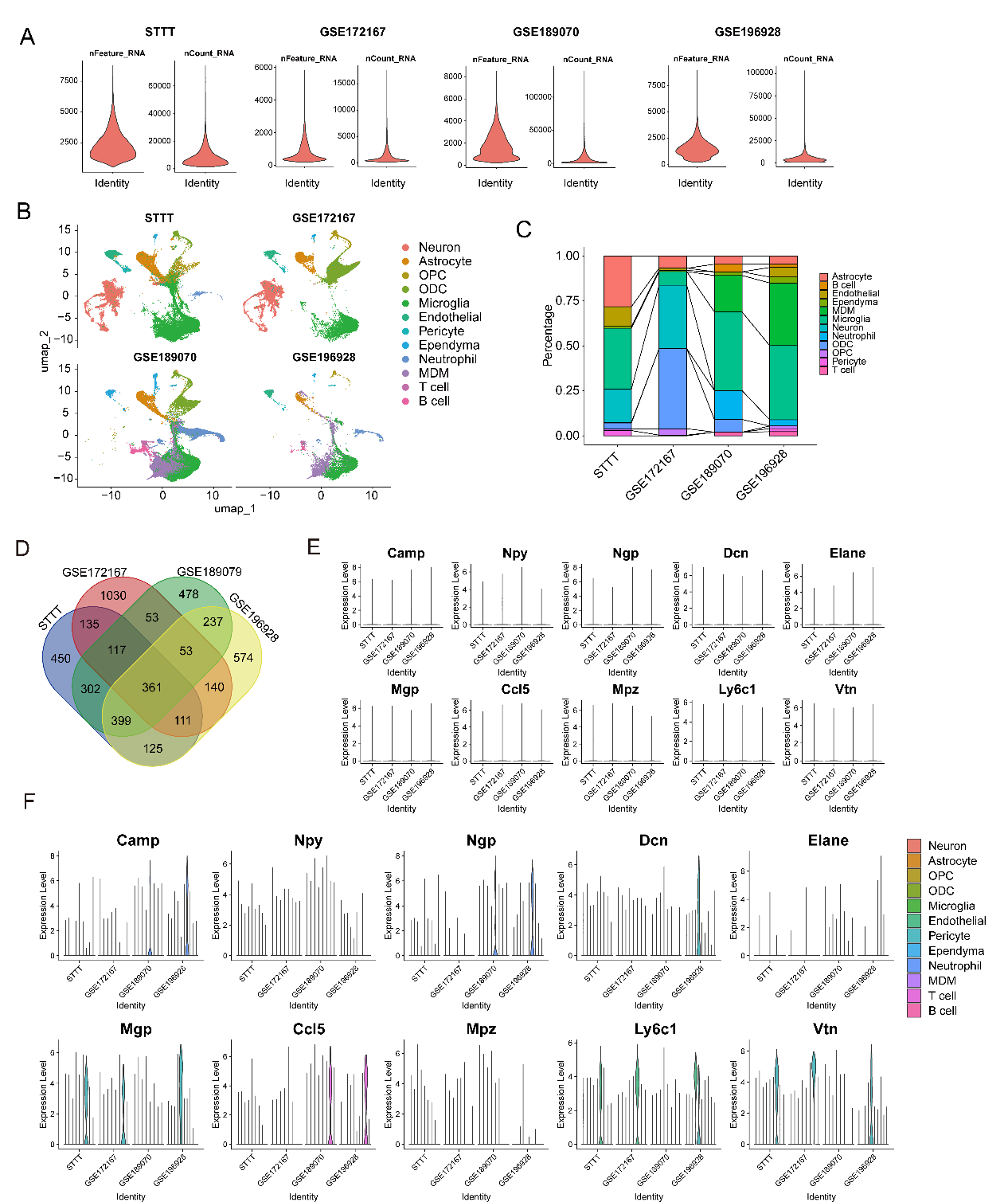


**Fig. S1. Comparative analysis of single-cell RNA sequencing datasets from different studies.** (A) Violin plots showing the distribution of the number of detected features (nFeature_RNA) and counts (nCount_RNA) for each dataset. (B) UMAP plots illustrating the clustering of major cell types identified across the datasets. (C) Stacked bar chart summarizing the proportion of different cell types identified in each dataset, showing consistency and variation across studies. (D) Venn diagram depicting the overlap of expressed genes across the four datasets, highlighting shared and dataset-specific gene expression patterns. (E) Expression profiles of selected marker genes (e.g., *Camp,* *Npy*, *Ngp*, *Dcn*, *Elane*, *Mgp*, *Ccl5*, *Mpz*, *Ly6c1*, and *Vtn*) in specific cell types across datasets, shown as violin plots. (F) Bar charts further illustrating the expression levels of the same marker genes in distinct cell types across the datasets, providing a detailed comparison of gene expression patterns.


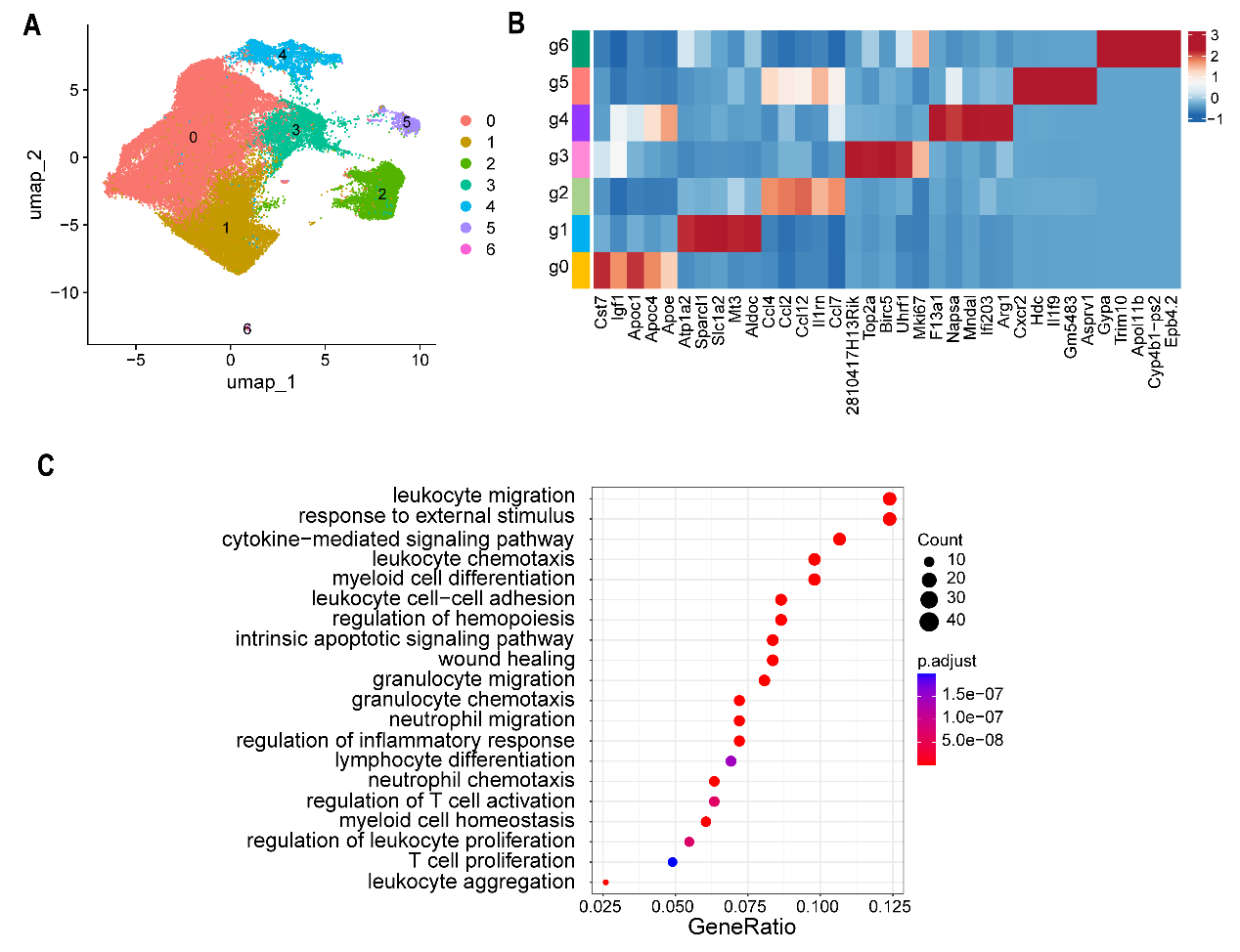


**Fig. S2. Microglia subpopulation subdivision and functional analysis.** (A) UMAP projections visualizing seven distinct microglial states were identified. (B) The heatmap shows the expression levels of selected marker genes across the identified clusters (0–6). (C) Dot plot represents the GO terms enriched in microglia at 1 dpi focusing on biological processes.


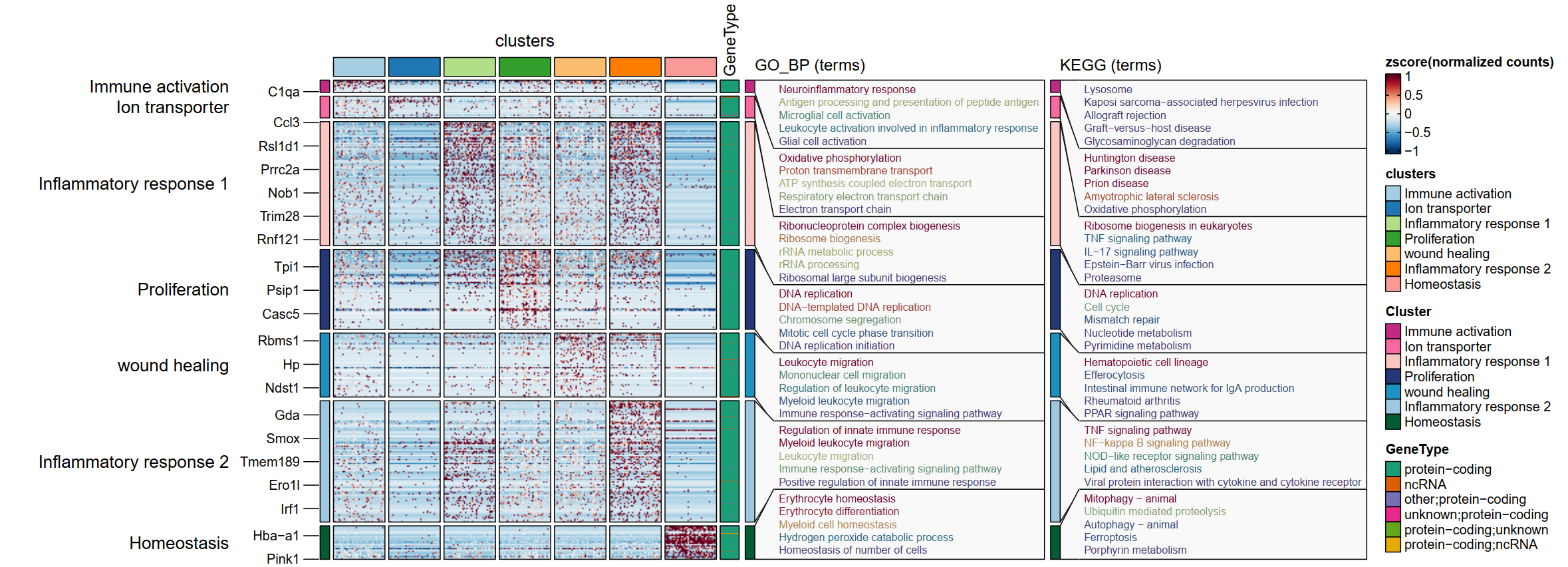


**Fig. S3. The enrichment analysis of microglia subcluster features includes GO and KEGG terms.** Subcluster of Inflammatory response enriched in inflammatory pathways including the NF-κB signaling pathway.


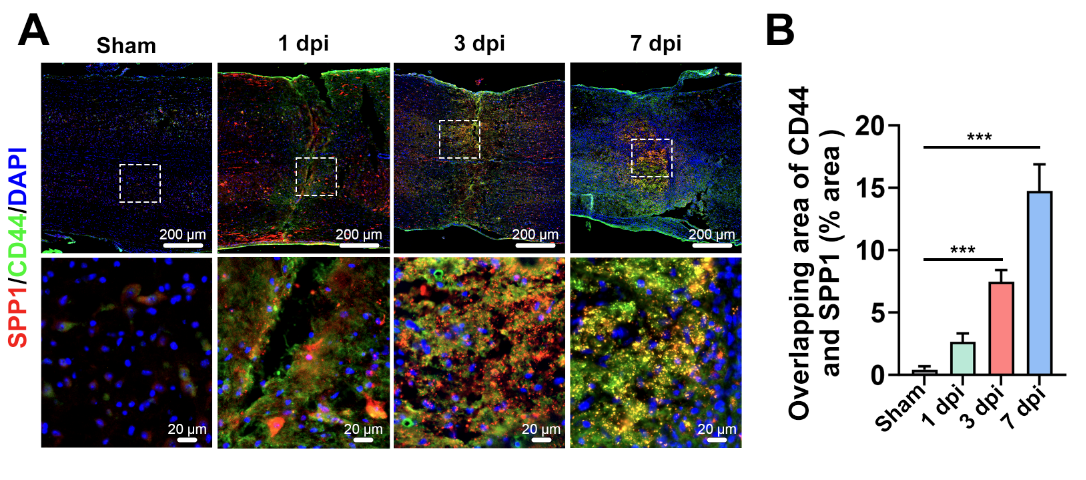


**Fig. S4. Spatial co-localization of SPP1 and CD44 in spinal cord sections after SCI.** (A) Representative immunofluorescence images showing CD44 (green), SPP1 (red), and DAPI (blue) co-staining in sham, 1 dpi, 3 dpi, and 7 dpi groups. Scale bar: 20 μm. (B) Quantitative analysis of SPP1-CD44 co-localization area expressed as percentage of total field area (% area). Data are presented as mean ± SEM (n = 3; ***P < 0.001).


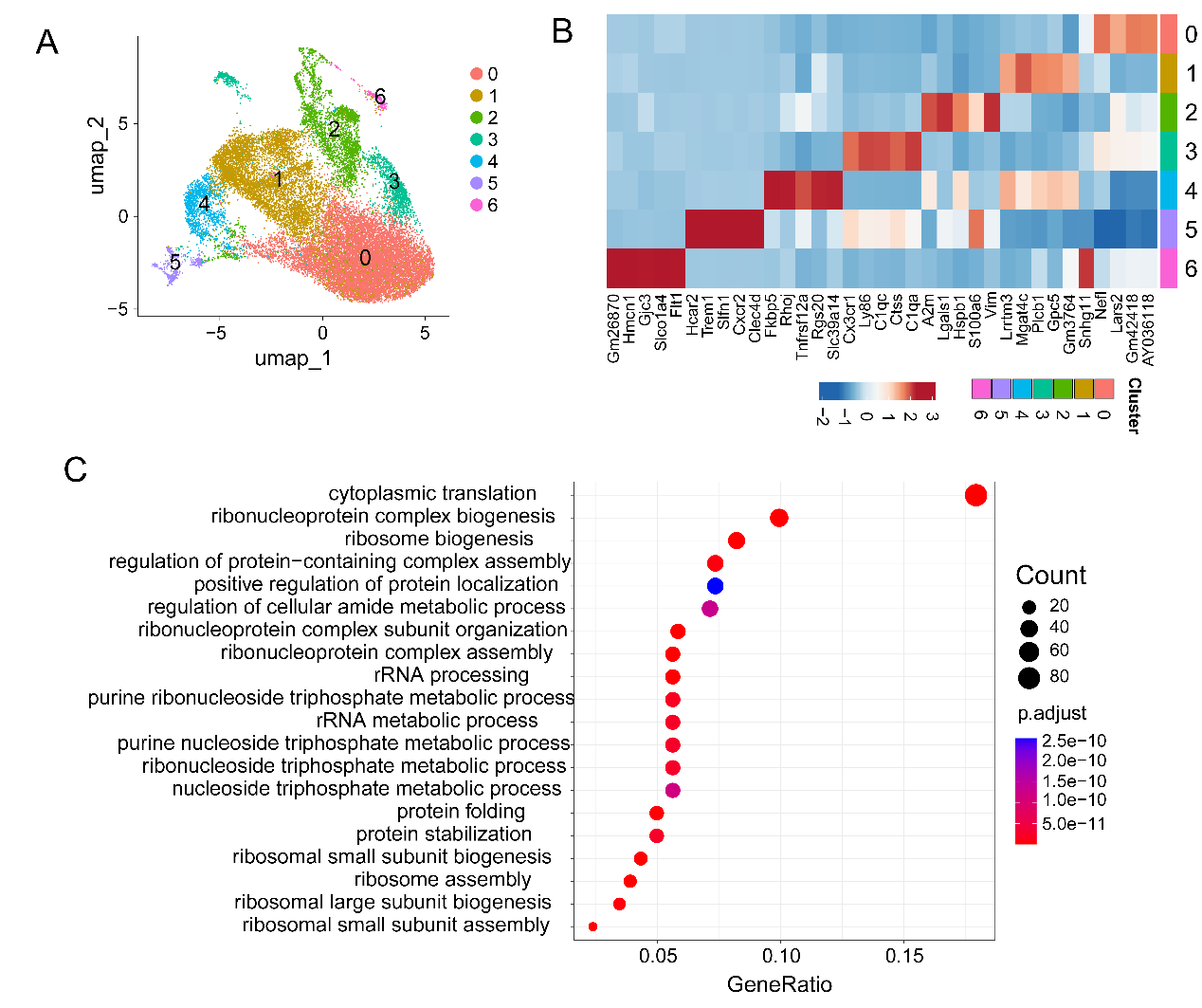


**Fig. S5. Astrocyte subpopulation subdivision and functional analysis.** (A) UMAP projections visualizing seven distinct astrocyte states were identified. (B) The heatmap shows the expression levels of selected marker genes across the identified astrocyte subclusters (0–6). (C) Dot plot represents the GO terms enriched in astrocyte at 3 dpi focusing on biological processes.


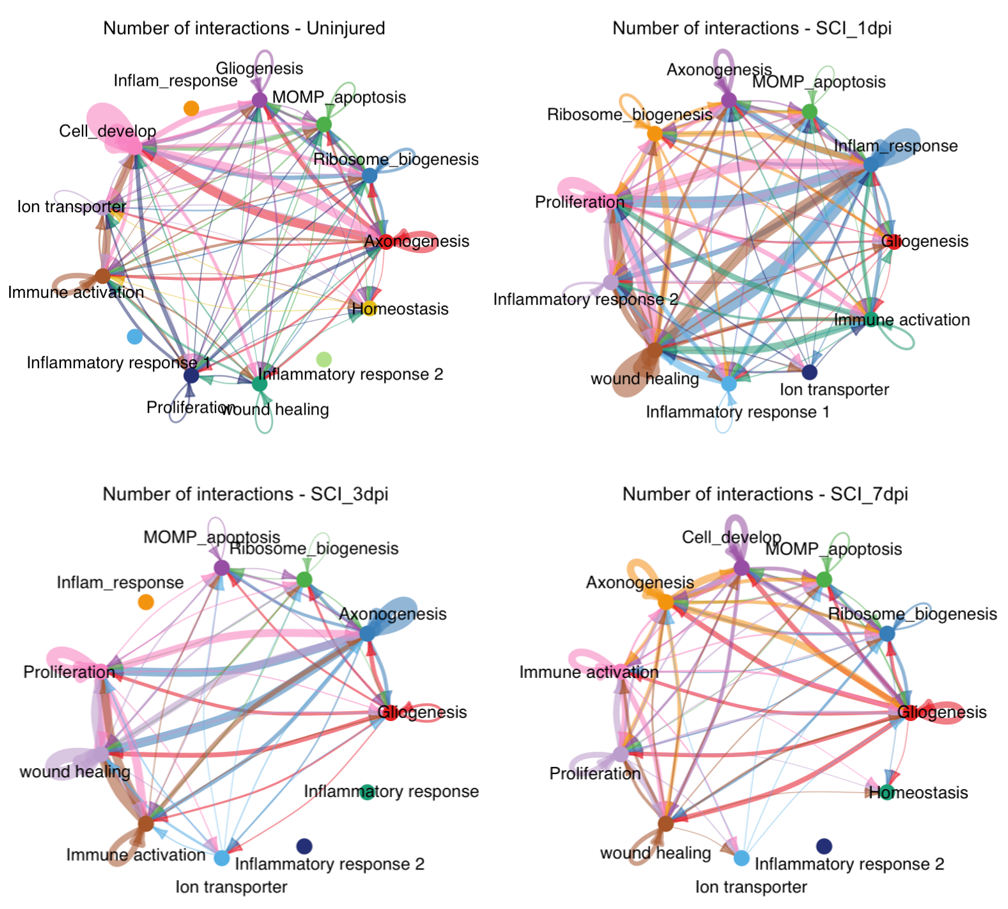


**Fig. S6. Analysis of cell communication between microglia subsets and astroglia subsets 7 days after injury.**


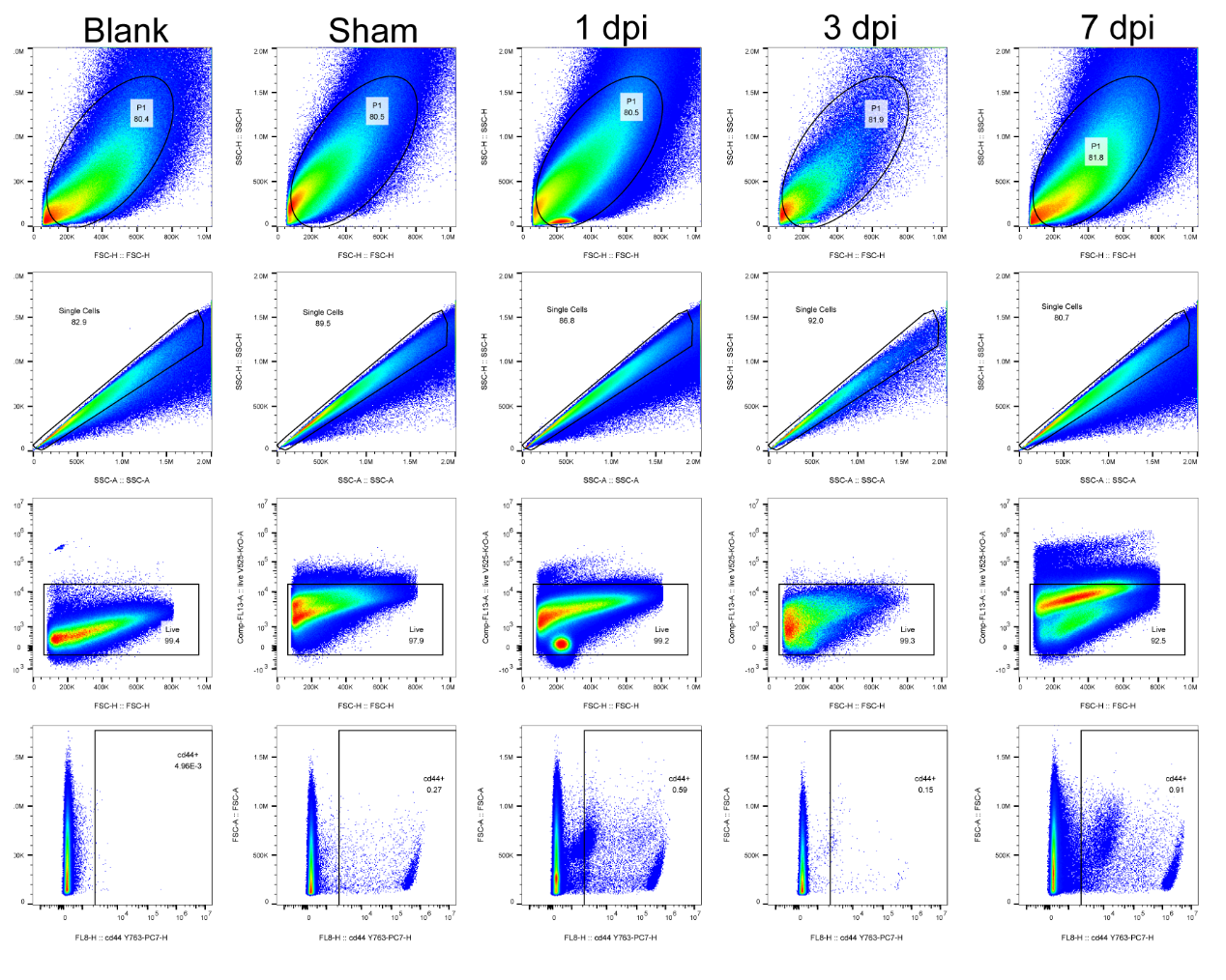


**Fig. S7. Flow cytometry results were used to identify live cells and CD44-positive microglia.**


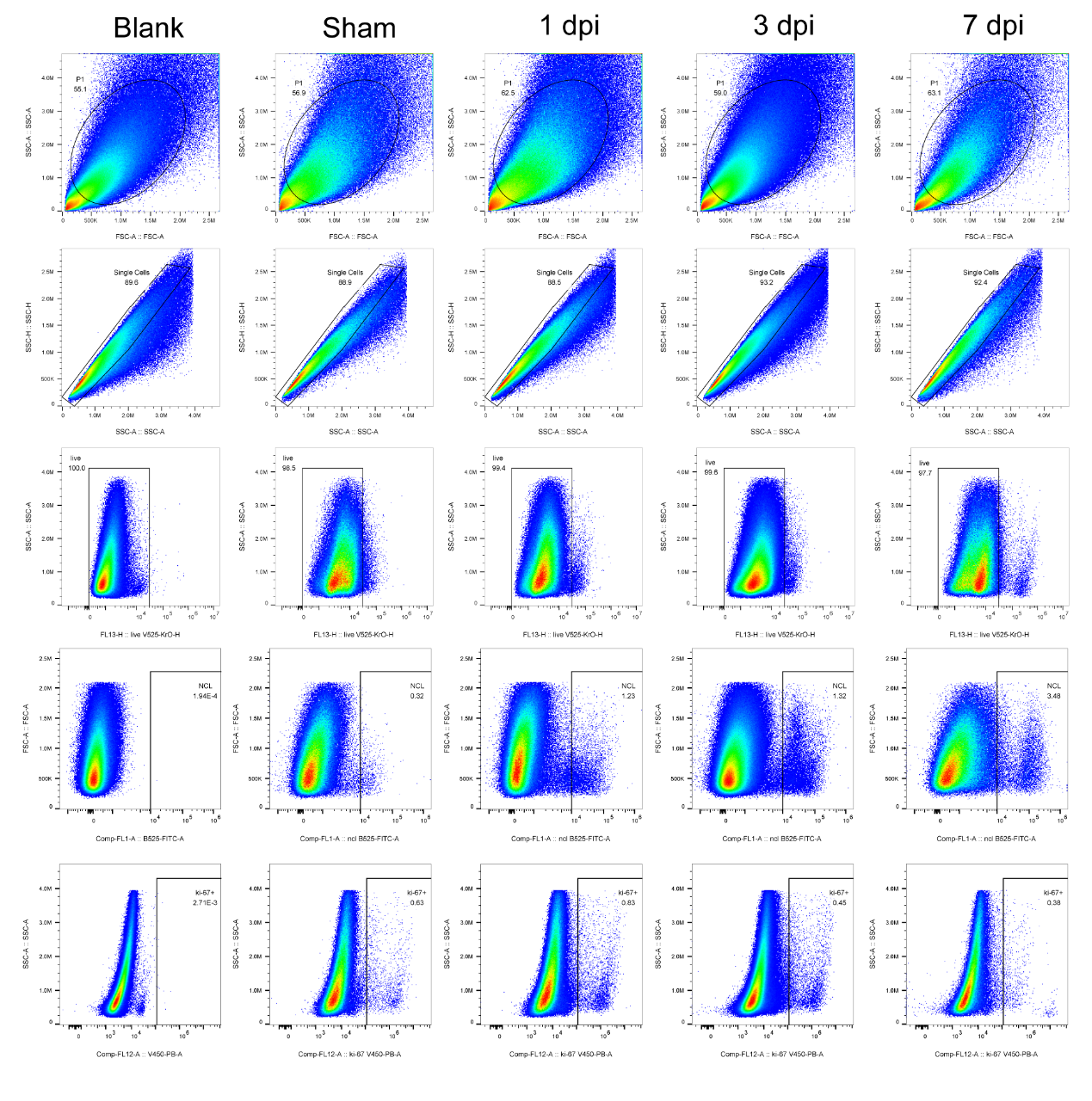


**Fig. S8. Flow cytometry results were used to determine the results of live cells, NCL, and Ki67 positive astrocytes.**


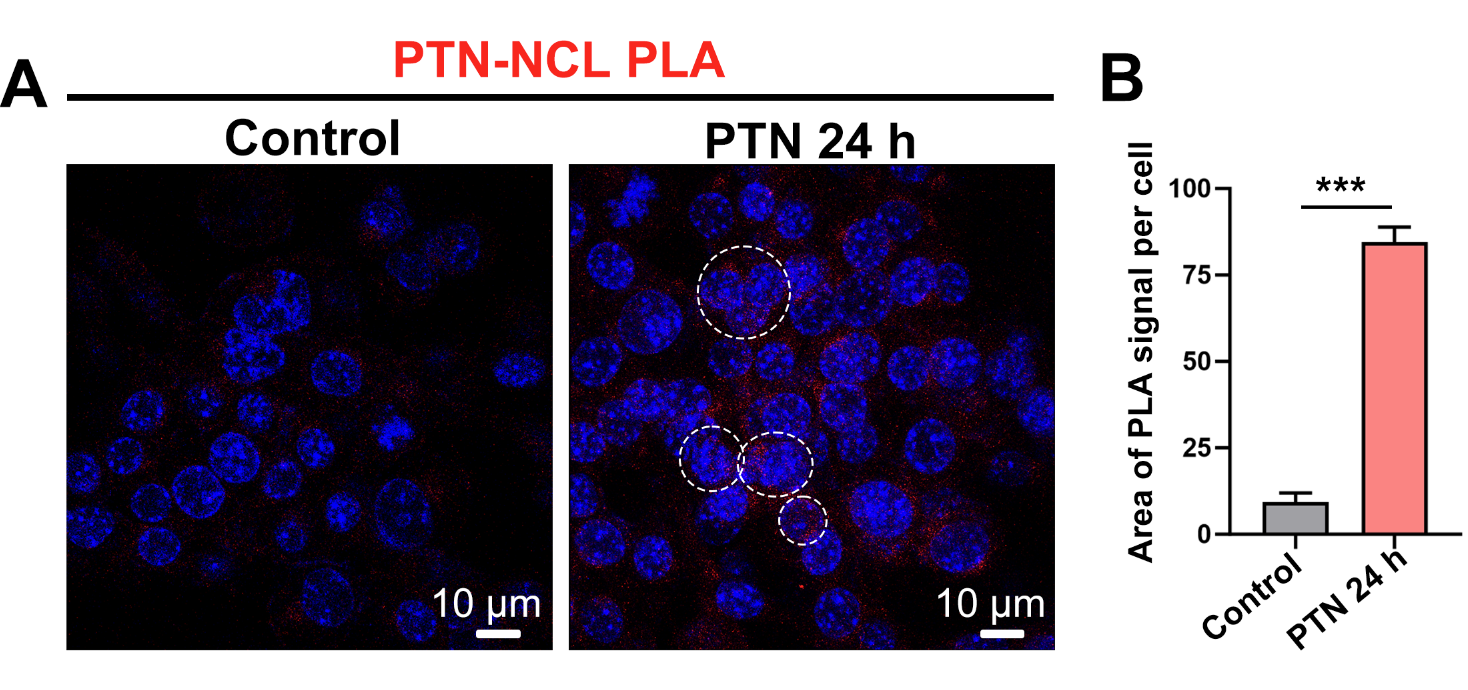


**Fig. S9. PTN interaction with NCL in astrocytes as detected by PLA.**(A) Representative images of PLA signals showing interaction between PTN and NCL in C8-D1A astrocyte cell under control condition and after PTN stimulation for 24 h. Nuclei were stained with DAPI. White circle indicated the PLA signal in nucleus, scale bar: 10 μm. (B) Quantification of PLA signal. Data was shown as area of PLA signal per cell which was calculated using the area of PLA signal per field/number of cells in the field. (n=3, ***P<0.001)
