# Supplementary material for: Integrative analysis and experimental validation identify the role of CD44 and Nucleolin in regulating gliogenesis following spinal cord injury
Source: Cell Regen. 2025 Aug 13;14:35. doi: 10.1186/s13619-025-00253-x (PMC12344060; doi:10.1186/s13619-025-00253-x)
Supplement: Supplementary file 2 — Supplementary Material 2: Table S1: A detailed description of the animal model in the four databases used in the integrated analysis. [file 13619_2025_253_MOESM2_ESM.docx]

Table S1. A detailed description of the animal model in the four databases used in the integrated analysis.

| Reference | Method | Species (Age) | Model | Tissue source |
| --- | --- | --- | --- | --- |
| Hou et al. (2022)  GSE189070 | scRNA-seq | Mouse (2-mth) | T10 right lateral over- hemisection | Uninjured and injured spinal cord at 0.5 dpi, 1 dpi, 3 dpi, 7 dpi, 14 dpi, 60 dpi, 90 dpi |
| Brennan et al. (2022)  GSE196928 | scRNA-seq | Mouse (8-10 weeks) | T9 contusion | Uninjured and injured spinal cord at 7 dpi and 28 dpi |
| Matson et al. (2022)  GSE172167 | snRNA-seq | Mouse12–30 weeks | T9 contusion | uninjured, 1 day, 1 week, 3 weeks , 6 weeks with 3 biological replicates |
| Li et al. (2022)  STTT | scRNA-seq | Mouse (8-10weeks) | T9 crush injury | Uninjured and injured spinal cord at 4 h, 1 d, 3 d, 7 d, 14 d, 28 d, 42 d |
